# The factors associated with mortality and progressive disease of nontuberculous mycobacterial lung disease: a systematic review and meta-analysis

**DOI:** 10.1038/s41598-023-34576-z

**Published:** 2023-05-05

**Authors:** Hyeontaek Hwang, Jung-Kyu Lee, Eun Young Heo, Deog Kyeom Kim, Hyun Woo Lee

**Affiliations:** 1 Division of Pulmonary and Critical Care Medicine, Department of Internal Medicine, Seoul National University Hospital, Seoul, Republic of Korea; 2 Division of Pulmonary and Critical Care Medicine, Department of Internal Medicine, Seoul National University College of Medicine, Seoul Metropolitan Government-Seoul National University Boramae Medical Center, 20, Boramae-ro 5-gil, Dongjak-gu, Seoul, 07061 Republic of Korea

**Keywords:** Infectious-disease epidemiology, Infectious diseases, Risk factors

## Abstract

This systematic review and meta-analysis aimed to comprehensively evaluate the factors associated with mortality and progressive disease in NTM-LD patients. We conducted a literature search to identify the eligible studies, dated between January 1, 2007, and April 12, 2021. Forty-one studies with total 10,452 patients were included. The overall all-cause mortality rate was 20% (95% CI 17–24%). The overall rates of clinical and radiographic progressive disease were 46% (95% CI 39–53%) and 43% (95% CI 31–55%), respectively. Older age, male sex, history of TB, diabetes, chronic heart disease, malignancy, systemic immunosuppression, chronic liver disease, presence of cavity, consolidative radiologic features, acid-fast bacillus (AFB) smear positivity, hypoalbuminemia, anemia, increasing platelet count, high CRP, and high ESR were significantly associated with increased all-cause mortality, whereas increasing body mass index (BMI), hemoptysis, and treatment with rifamycin regimen (in *M. xenopi*) were significantly associated with decreased all-cause mortality in multivariable analysis. History of TB, Aspergillus co-infection, cough, increased sputum, weight loss, presence of cavity, and AFB smear positivity were significantly associated with increased clinical progression with treatment, while older age and low BMI were significantly associated with decreased clinical progression in multivariable analysis. Older age, interstitial lung disease, presence of cavity, consolidative radiologic feature, anemia, high CRP, and leukocytosis were significantly associated with increased radiographic progression after adjusting for covariates. Older age, history of tuberculosis, presence of cavity, consolidative radiologic features, AFB smear positivity, anemia, and high C-reactive protein were common significant factors associated with the all-cause mortality and clinical or radiographic progressive disease of NTM-LD. These factors are thought to directly affect NTM-LD related mortality. The future prediction models for the prognosis of NTM-LD should be established considering these factors.

## Introduction

Nontuberculous mycobacterial lung disease (NTM-LD), the most common clinical manifestation of NTM is diagnosed based on clinical, radiographic, and microbiological criteria^1,2^. The incidence and prevalence of NTM-LD have been increasing worldwide^3^. Despite the increasing burden, previous studies have reported unsatisfactory success rates for antibiotic therapy for the disease^4,5^. A barrier to effective treatment is the highly heterogeneous clinical course of NTM-LD and there have been numerous attempts at identifying the clinical variables contributing to a better prognosis^1,6^.

Identifying factors associated with mortality and progressive disease in NTM-LD is important as it will facilitate effective treatment decisions by physicians and aid researchers in designing future intervention studies^7^. The decision to start antibiotic therapy for NTM-LD is currently based on disease severity or risk factors for progressive disease and mortality^8^. However, there is a lack of randomized clinical trials (RCTs) that address when NTM-LD treatment should be started and that information is based on observational data. Many observational studies have been conducted to identify the factors associated with mortality and progressive disease in NTM-LD; however, results vary depending on the study settings^9,10^. Moreover, evidence of these factors remains inconclusive as most of the research is underpowered^11,12^.

This systematic review and meta-analysis aimed to comprehensively evaluate the factors associated with mortality and progressive disease and assess their effect sizes, in NTM-LD patients.

## Results

### Study selection

**Figure 1:**
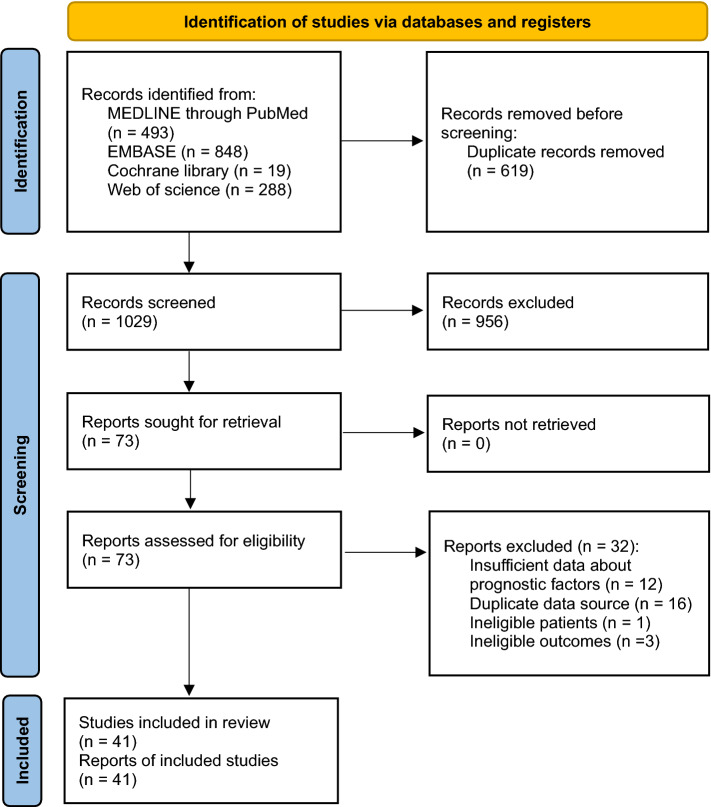
Preferred Reporting Items for Systematic Review and Meta-analysis (PRISMA) flow diagram for the systematic review and meta-analysis.

A total of 1648 records were identified using the Medline, Embase, Cochrane Central Register of Controlled Trials, and Web of Science databases. No additional records from other sources were included. After the removal of duplicate studies, 1029 studies were screened and 73 potentially eligible studies were retrieved for the full-text review. Finally, 41 studies satisfied the eligibility criteria (Fig. 1). The reasons for excluding the 32 studies are described in Appendix S1. Of the 41 studies, 40 were observational studies and one was an RCT. Of the observational studies, 34 were retrospective, four were prospective, and two had data from both the prospective and retrospective cohorts.

### Baseline characteristics of the included studies and patients

A total of 41 studies were included in this review, which evaluated factors associated with progressive disease and death in 10,452 patients (Table 1). Thirty-four studies were conducted in Asia, four studies were conducted in Europe, and three were conducted in North America. Most studies were conducted in Japan (n = 21), followed by South Korea (n = 9). The mean age was 64.9 years and 36.4% were men. *M. avium* complex (MAC) infection was the most common (85.1%), followed by *M. abscessus* (7.8%). In the radiologic pattern, 69.6% of the patients had nodules, bronchiectasis, or nodular bronchiectatic pattern, 17.5% had a cavity or fibrocavitary pattern, and 0.7% had consolidation.

**Table 1:** Characteristics of 41 included studies.

No.	First author (published year)	N^†^	Study period	Country	Study design	Age, mean^†^	Male, %^†^	Inclusion criteria	Microbiologic features, %^†^	Radiologic features, %^†^	Antibiotic treatment, %‡	Outcome, %^$^	Mean duration of follow up, month
1	Abate, G. et al	221 (297)	2005–2015	United States	Retrospecitve cohort	(64*)	31.7 (37.4)	Adult patients diagnosed with NTM-PD without concurrent HIV or TB infection	MAC: 100 (83.2) M. kansasii: 0 (7.7) MAB: 0 (4.0)	Nodules: 56.1 (52.2) BE: 48.0 (43.1) Cavity: 23.5 (26.9)	81.4 (82.5)	Death: 15.4	24.2*
2	Akahori, D. et al	248	Not described	Japan	Retrospective cohort	74.3	39.5	MAC-LD patients with CT and BMI measurement for ≥ 6 months	MAC: 100	NB: 76.2 FC: 23.8	57.7	Death: 17.7	61.3
3	Andréjak, C. et al	122 (136)	1983–2003	France	Retrospective cohort	(53)	(76.5)	Adult patients with M. xenopi pulmonary infection	M. xenopi: 100	Nodular: (30.1) Cavity: (28.7) Consolidation: (33.1)	60.7 (58.8)	Death (within 3 years): 65.6	Not described
4	Asakura, T. et al. (2017)	125	1994–2015	Japan	Retrospective cohort	60*	47	Patients with NTM-PD treated by pulmonary resection	MAC: 88.0 MAB: 4.8 M. kansasii: 3.2	Nodule: 59.2 BE: 51.2 Cavity: 12.8	93.6	Death: 20.8	85.2*
5	Asakura, T. et al. (2021)	187 (281)^&^	2012–2016	Japan	Prospective cohort	68.4	20.9	Adult MAC-LD patients without current treatment and other diseases for elevating KL-6	MAC: 100	NB: 77.0 FC: 3.2	Not applicable	Death: 10.0 Clinical progressive disease: 28.3	64.6
6	Chang, C. L. et al	144	2013–2015	Taiwan	Retrospective cohort	70.8*	44.4	Patients diagnosed with MAC-PD	MAC: 100	NB: 75.0 FC: 12.5	22.9	Radiographic progressive disease (within 2 years): 10.4	24
7	Fleshner, M. et al	106	2010–2011	United States	Retrospective cohort	55*	13.2	Adult HIV negative patients with PNTM without cystic fibrosis	MAC: 61.3 MAB: 34.9	FC: 45.5	100	Death: 25.5	58.8*
8	Fukushima, K. et al	295	2008–2019	Japan	Retrospective cohort	65.9	23.7	Adult MAC-PD patients with first-line antibiotic therapy for ≥ 6 months	MAC: 100	NB: 84.4 Cavity: 43.1	100	Death:16.3	Not described
9	Gochi, M. et al	536 (782)^#^	1999–2010	Japan	Retrospective cohort	(68.1)	(31.5)	Adult HIV-negative patients newly diagnosed with NB MAC-LD	MAC: 100	NB: 100	(30.8)	Death: 16.6 Radiographic progressive disease: 41.2	Death: 51.6* Progressive disease: 60.0*
10	Hachisu, Y. et al	150	2014–2018	Japan	Retrospective cohort	70.0*	34.0	Adult patients diagnosed with NTM-LD	MAC: 88.0 M. kansasii: 1.3	NB: 66.7 FC: 8.0	Not described	Death: 11.3	24*
11	Hong, J. Y. et al	89	2007–2015	South Korea	Case–control	71*	44.9	PNTM patients followed up for at least 1 year with chest CT and laboratory tests	MAC: 69.7 MAB: 12.4 M. kansasii: 2.2	BE: 95.5 Cavity: 40.4	49.4	Radiographic progressive disease: 41.6	26*
12	Hwang, J. A. et al	93 (420)	1998–2011	South Korea	Retrospective cohort	(62*)	(51.2)	Patients newly diagnosed with MAC-LD followed up for ≥ 3 years	MAC: 100	NB: (56.0) FC: (35.2)	0 (77.9)	Death: 17.2	Death: 76.8* Progressive disease: 43.2*
13	Inomata, T. et al	22 (37)	2000–2014	Japan	Retrospective cohort	67* (65*)	18.2 (10.8)	Immunocompetent patients diagnosed with NB pulmonary MAC infection without concomitant other pulmonary diseases	MAC: 100	NB: 100	0 (16.2)	Radiographic progressive disease (within 1 year): 31.8	11.3
14	Ito, Y. et al	78 (164)	1999–2005	Japan	Retrospective cohort	65.2 (66.0)	39.7 (43.3)	Patients diagnosed with pulmonary MAC disease not based on surgical specimens from unknown solitary nodules	MAC: 100	BE: 59.0 (48.8) Cavity: 25.6 (18.3)	69.2 (42.7)	Death (within 5 years): 25.6	60
15	Jenkins, P. A. et al	371	1995–1999	8 countries in Europe	Randomized controlled trial	64.1	58.5	HIV-negative adult patients diagnosed with NTM-LD without possibility of pregnancy	MAC: 45.8 M. malmoense: 45.0 M. xenopi: 9.2	Cavity: 56.3	100	Death (within 5 years): 43.1	60
16	Jhun, B. W. et al	1445	1997–2013	South Korea	Prospective cohort plus retrospective cohort	60*	39	Patients newly diagnosed with MAC or MAB-PD (excluding M. bolletii)	MAC: 79.0 MAB: 21.0	NB: 82.1 FC: 17.9	61.8	Death: 19.6	77.6*
17	Kadota, T. et al	33	2009–2013	Japan	Retrospective cohort	67	6.1	Patients newly diagnosed with clarithromycin-resistant MAC-PD and treated for ≥ 5 months after diagnosis	MAC: 100	NB: 24.2 FC: 75.8	100	Radiographic progressive disease (within 1 year): 54.5	10.4*
18	Kang, H. R. et al.**	421	2007–2018	South Korea	Retrospective cohort	64*	35.6	Adult NTM-PD patients followed up for ≥ 1 year with chest CT scans at the time of diagnosis	MAC: 71.0 MAB: 19.5	Cavity: 21.9	43.2	Death: 7.6	49*
19	Kikuchi, T. et al	24 (26)	2005–2006	Japan	Retrospective cohort	57 (58)	20.8 (26.9)	Patients diagnosed with M. avium LD	M. avium: 100	NB: 87.5 (84.6) FC: 12.5 (11.5)	Not applicable	Clinical progressive disease (within 1 year): 62.5	Not described
20	Kim, H. J. et al	347	2011–2017	South Korea	Prospective cohort	66*	38.0	Adult untreated NTM-PD patients without poor compliance (delaying visits for > 1 month)	MAC: 70.0 MAB: 18.2 M. kansasii: 2.0	NB: 90.8 Cavity: 9.2	Not applicable	Clinical progressive disease: 38.3	34.8*
21	Kim, S. J. et al	67	2004–2013	South Korea	Retrospective cohort	60.3	31.3	NB MAC-LD patients who had undergone chest CT with at least 18-month intervals and been followed up > 5 years	MAC: 100	NB: 100	52.2	Radiographic progressive disease: 50.7	80*
22	Kodaka, N. et al	167 (238)	2011–2018	Japan	Retrospective cohort	78* (76*)	34.7 (31.9)	Patients newly diagnosed with pulmonary MAC disease	MAC: 100	NB: Not described (80.7) FC: 14.4 (19.3)	0	Radiographic progressive disease (within 1 year): Not described	12
23	Kumagai, S. et al	368 (486)	2006–2011	Japan	Retrospective cohort	72	41.0 (40.3)	Adult HIV-negative patients newly diagnosed with MAC-LD	MAC: 100	NB: 81.0 (82.1) FC: 11.1 (11.3)	45.9 (48.4)	Death: 20.4	42*
24	Kwon, B. S. et al	551	2000–2013	South Korea	Retrospective cohort	61.1	32.8	Patients with NC-NB MAC-LD, diagnosed by CT, and a follow-up duration greater than 3 years	MAC: 100	NB: 100	Not applicable	Clinical progressive disease (within 3 years):58.6	69.6*
25	Liu, C. J. et al	109	2010–2014	Taiwan	Retrospective cohort	69.2	65.1	Patients newly diagnosed with MK-PD followed up for 1 year with chest radiography	M. kansasii: 100	FC: 38.5	3.7	Radiographic progressive disease (within 1 year): 64.2	12
26	Matsuda, S. et al	229	2013–2015	Japan	Retrospective cohort	71*	30	Newly diagnosed MAC-LD patients with anti-GPL-core IgA antibody measurements without concurrent bacterial pneumonia or TB infection	MAC: 100	NB: 80.8 FC: 7.0	Not applicable	Clinical progressive disease: 49.8	12.1*
27	Moon, S. M. et al	1021	2003–2013	South Korea	Prospective cohort plus retrospective cohort	59*	32	Patients newly diagnosed with NC-NB MAC-PD or MAB-PD (excluding M. bolletii)	MAC: 80.6 MAB: 19.4	NB: 100	Not applicable	Clinical progressive disease: 55.0	26.7
28	Moon, S. W. et al	663	2005–2017	South Korea	Retrospective cohort	64.1	44.0	Newly diagnosed HBV-, HIV-negative MAC-PD patients with CT images at the time of diagnosis	MAC: 100	Cavity: 23.4	46.2	Death: 9.5	47.3
29	Mori, S. et al	225	2009–2018	Japan	Retrospective cohort	70.7	29.3	Adult patients newly diagnosed with PNTM disease (15% of patients had rheumatoid arthritis)	MAC: 90.7 MAB: 6.7	NB: 71.6 FC: 12.0	45.3	Death: 27.1	47.5
30	Moriyama, M. et al	46	2008–2009	Japan	Retrospective cohort	67.1	34.8	Untreated patients diagnosed with pulmonary M. avium disease	M. avium: 100	NB: 56.5 FC: 37.0	Not applicable	Clinical progressive disease (within 18 months): 37.0	18
31	Naito, M. et al	62	2010–2015	Japan	Retrospective cohort	69.5*	61.3	Patients with chronic pulmonary aspergillosis and a history of PNTM	MAC: 59.7 M. kansasii: 32.3	NB: 40.3 FC: 40.3	83.9	Death: 35.5	50.4*
32	Ogawa, T. et al	269	2012–2018	Japan	Prospective cohort	68*	19.3	Adult MAC-PD patients who completed the St George’s Respiratory Questionnaire	MAC: 100	NB: 71.7 FC: 3.7	52.8	Death: 8.2	49.2*
33	Oshitani, Y. et al	97	2006–2016	Japan	Retrospective cohort	65.7	17.5	MAC-PD patients with cavities observed for > 3 years and evaluated with CT at two or more points	MAC: 100	Nodules: 62.9 BE: 66.0 Cavity: 100	84.5	Radiographic progressive disease: 53.6	100.5
34	Provoost, J. et al	51	2013–2016	France	Retrospective cohort	68*	43.1	Adult NTM-LD patients without systemic immunodeficiency	MAC: 78.4 M. xenopii: 17.6 M. simiae: 5.9	Nodules: 93.8 BE: 52.1 Cavity: 54.2	Not applicable	Clinical progressive disease: 49.0	17.1*
35	Raats, D. et al	272 (497)	2003–2015	Canada	Retrospective cohort	63.2* (65.9*)	35.3 (35.8)	NTM-PD patients who had a respiratory sample submitted for fungal culture	MAC: 71.7 (72.6) M. xenopi: 15.1 (15.3) M. abscessus: 7.0 (5.6)	NB: 61.8 (65.0) FC: 23.9 (16.9)	100 (65.8)	Death: 19.1 (15.7) Radiographic progressive disease (within 1 year): 25.6 (Not described)	46.0*
36	Rawson, T. M. et al	102 (108)	2010–2014	United Kingdom	Retrospective cohort	(68*)	(61)	HIV-negative patients with pulmonary NTM disease without concomitant TB	MAC: (50.9) M. kansasii: (15.7)	NB: (32.4) Cavity: (26.9)	Not applicable	Clinical progressive disease: 39.2	Not described
37	Shirai, T. et al	131	2007–2014	Japan	Retrospective cohort	72*	42.7	Newly diagnosed MAC-LD patients checked for aspergillus precipitating antibody at the time of diagnosis	MAC: 100	NB: 58.0 FC:26.7	72.5	Death: 26.0	48*
38	Shu, C. C. et al	252 (481)	2007–2009	Taiwan	Retrospective cohort	63.7	50.8	Patients diagnosed with NTM-LD	MAC: 39.7 M. chelonae-abscessus: 30.2 M. kansasii: 11.1	BE: 53.2 Cavity: 24.6 Consolidation: 22.2	40.1	Death (within 6 months): 9.9	Not described
39	Ushiki, A. et al	26	Not described	Japan	Prospective cohort	67	11.5	Patients with pulmonary MAC infection	MAC: 100	NB: 34.6 Cavity: 30.8 Consolidation: 34.6	0	Radiographic progressive disease: 57.7	11
40	Wang, P. H. et al	123	2011–2017	Taiwan	Retrospective cohort	66.7	51.2	Newly diagnosed MAC-LD patients without co-infection of TB or HIV	MAC: 100	NB: 61.0 FC: 15.4	38.2	Death (within 4 years): 17.9	49.8
41	Yamamoto, Y. et al	224	2012–2016	Japan	Retrospective cohort	67.0	22.3	Newly diagnosed MAC-LD patients who underwent high-resolution CT	MAC: 100	NB: 77.2 FC: 21.4	Not applicable	Clinical progressive disease (within 3 years): 46.0	Not described

BE, bronchiectasis; BMI, body mass index; CT, computed tomography; FC, fibrocavitary; GPL, glycopeptidolipid; HBV, hepatitis B virus; HIV, human immunodeficiency virus; IgA, immunoglobulin A; KL-6, Krebs von den Lungen-6; LD, lung disease; MAB, Mycobacterium abscessus; MAC, *Mycobacterium avium* complex; MK, *Mycobacterium kansasii*; NB, nodular/bronchiectatic; NC, non-cavitary; NTM, nontuberculous mycobacteria; PD, pulmonary disease; PNTM, pulmonary nontuberculous mycobacteria; TB, tuberculosis.

^†^Some studies evaluated the association between factors and outcomes only in the subgroup of patients with NTM-LD. If the information is available, we described the information of the subgroup patients first, and then the information of all patients in the bracket.

^‡^This means the proportion of antibiotics treated before the outcome event occurred. If the outcome was clinical progressive disease, it was described as not applicable.

^$^Clinical progressive disease is defined as anti-NTM treatment is required or initiated. Radiographic progressive disease is defined as radiographic deterioration or worsening. Except for some studies, outcomes occurred during the total follow-up period of the studies.

*Only median value was reported.

^#^Analysis of death was performed in all 782 patients. Of 782 patients, an analysis of radiographic progressive disease was performed in 536 patients.

^&^Analysis of death was performed on all 281 patients. Of 281 patients, an analysis of radiographic progressive disease was performed in 187 patients.

**We extracted data about > 2 cm sized cavity not including data about ≤ 2 cm sized cavity as variable ‘presence of cavity’ in Kang, H. R. et al.

### Risk of bias assessment

The risk of bias (ROB) assessment for all included studies is described in Appendix S2. Most studies had high ROBs for study attrition due to lack of information on the sample attrition. Moreover, there were moderate-to-high ROBs in the study participation and adjustment for confounders, which was due to the low participation rate, selective enrollment in the study, and insufficient consideration for other factors.

### All-cause mortality

**Figure 2:**
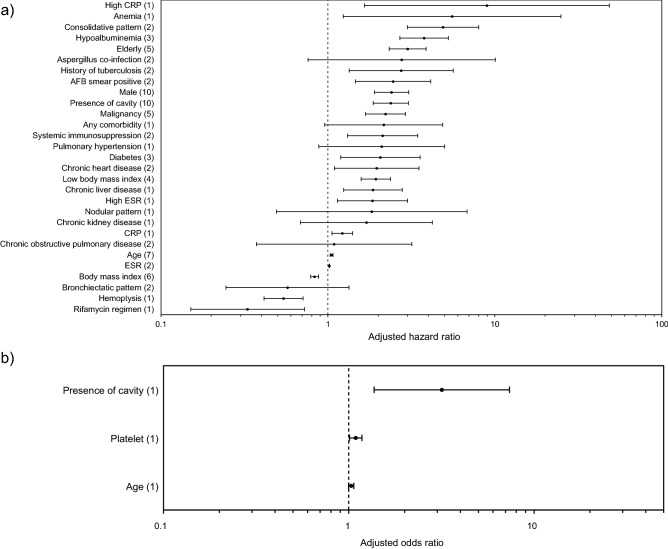
Forest plots displaying the pooled hazard ratios estimating the association between factors and all-cause mortality. (**a**) Analysis of adjusted hazard ratios. (**b**) Analysis of adjusted odds ratios. The numbers within the parentheses mean the numbers of studies analyzed. All adjusted risk ratios were estimated in multivariable analysis.

A total of 7,103 patients from 23 studies were included in the review of factors associated with all-cause mortality^9,11,13–33^. The pooled adjusted RRs and unadjusted RRs, which estimated the association between factors and all-cause mortality, were visualized by forest plots (Fig. 2 and Figure S1). The pooled risk ratios for each factor are presented as a forest plot in Appendix S3. The factors significantly associated with all-cause mortality and progressive disease in univariable analysis were summarized in Appendix S4. Categorical variables and continuous variables were separately analyzed for the association with all-cause mortality and progressive disease. The definition of some categorical variables defined differently in each study is outlined in Appendix S5.

The overall rate of all-cause mortality was 20% (95% CI 17–24%). The heterogeneity of the effect estimate (I^2^) was 94.8%. The factors significantly associated with all-cause mortality, clinical or radiographic progressive disease were summarized in Table 2. Multivariable analysis showed that increasing age (adjusted HR = 1.052; 95% CI 1.031–1.074; I^2^ = 19.6%; seven studies), the elderly (adjusted HR = 3.005; 95% CI 2.329–3.876; I^2^ = 22.6%; five studies), male (adjusted HR = 2.406; 95% CI 1.900–3.047; I^2^ = 46.3%; ten studies), and low body mass index (BMI) (adjusted HR = 1.934; 95% CI 1.581–2.366; I^2^ = 0; four studies) had a significant association with increased all-cause mortality, while increasing BMI (adjusted HR = 0.832; 95% CI 0.788–0.878; I^2^ = 0; six studies) was significantly associated with decreased all-cause mortality.

**Table 2:** The factors significantly associated with all-cause mortality, clinical or radiographic progressive disease in multivariable analysis.

Factors	All-cause mortality		Clinical progressive disease		Radiographic progressive disease	
Age	↑	aHR 1.052 (1.031–1.074)	↓	aHR 0.976 (0.967–0.985) aOR 0.950 (0.920–0.980)	↑	aOR 1.120 (1.038–1.209)
Elderly	↑	aHR 3.005 (2.329–3.876)	N/A	N/A	↑	aOR 2.980 (1.041–8.534)
Male	↑	aHR 2.406 (1.900–3.047)	X	N/A	X	N/A
Body mass index	↓	aHR 0.832 (0.788–0.878)	X	N/A	X	N/A
Low body mass index	↑	aHR 1.934 (1.581–2.366)	↓	aOR 0.515 (0.310–0.855)	X	N/A
Ever-smoking	N/A	N/A	N/A	N/A	N/A	N/A
Any comorbidity	X	N/A	N/A	N/A	N/A	N/A
Diabetes	↑	aHR 2.062 (1.194–3.562)	X	N/A	X	N/A
Chronic lung disease	N/A	N/A	N/A	N/A	N/A	N/A
Chronic obstructive pulmonary disease	X	N/A	X	N/A	N/A	N/A
History of tuberculosis	↑	aHR 2.749 (1.341–5.637)	↑	aHR 1.230 (1.009–1.499)	X	N/A
Bronchiectasis	N/A	N/A	N/A	N/A	N/A	N/A
Asthma	N/A	N/A	N/A	N/A	X	N/A
Interstitial lung disease	N/A	N/A	N/A	N/A	↑	aHR 2.191 (1.325–3.623)
Pulmonary hypertension	X	N/A	N/A	N/A	N/A	N/A
Chronic heart disease	↑	aHR 1.959 (1.093–3.509)	N/A	N/A	X	N/A
Chronic liver disease	↑	aHR 1.860 (1.242–2.785)	N/A	N/A	N/A	N/A
Chronic kidney disease	X	N/A	N/A	N/A	N/A	N/A
Malignancy	↑	aHR 2.213 (1.680–2.914)	X	N/A	N/A	N/A
Systemic immunosuppression	↑	aHR 2.126 (1.311–3.450)	N/A	N/A	N/A	N/A
HIV	N/A	N/A	N/A	N/A	X	N/A
Aspergillus co-infection	X	N/A	↑	aOR 5.330 (1.107–25.662)	N/A	N/A
Hemoptysis	↓	aHR 0.542 (0.414–0.709)	X	N/A	N/A	N/A
Cough	N/A	N/A	↑	aHR 1.360 (1.053–1.756)	N/A	N/A
Sputum	N/A	N/A	↑	aHR 1.470 (1.131–1.911)	N/A	N/A
Fatigue	N/A	N/A	N/A	N/A	N/A	N/A
Dyspnea	N/A	N/A	N/A	N/A	N/A	N/A
Fever	N/A	N/A	N/A	N/A	N/A	N/A
Chest pain	N/A	N/A	N/A	N/A	N/A	N/A
Weight loss	N/A	N/A	↑	aOR 2.822 (1.271–6.268)	N/A	N/A
Nodular pattern	X	N/A	N/A	N/A	N/A	N/A
Bronchiectatic pattern	X	N/A	N/A	N/A	N/A	N/A
Nodular-bronchiectatic pattern	N/A	N/A	N/A	N/A	N/A	N/A
Presence of cavity	↑	aHR 2.380 (1.866–3.037) aOR 3.176 (1.369–7.369)	↑	aHR 3.460 (2.273–5.267) aOR 5.324 (2.323–12.199)	↑	aHR 1.651 (1.181–2.308) aOR 3.283 (1.405–7.673)
Consolidative pattern	↑	aHR 4.895 (2.997–7.996)	N/A	N/A	↑	aOR 16.150 (4.048–64.429)
M. avium complex	N/A	N/A	N/A	N/A	N/A	N/A
M. abscessus	N/A	N/A	N/A	N/A	X	N/A
M. kansasii	N/A	N/A	N/A	N/A	N/A	N/A
M. xenopi	N/A	N/A	N/A	N/A	N/A	N/A
Rifamycin regimen	↓	aHR 0.330 (0.151–0.723)	N/A	N/A	N/A	N/A
Treatment duration	N/A	N/A	N/A	N/A	N/A	N/A
Treatment with 3 or more antibiotics	N/A	N/A	N/A	N/A	N/A	N/A
AFB smear positivity	↑	aHR 2.456 (1.460–4.130)	↑	aOR 2.132 (1.393–3.263)	X	N/A
WBC	N/A	N/A	N/A	N/A	N/A	N/A
Leukocytosis	N/A	N/A	N/A	N/A	↑	aOR 3.440 (1.139–10.390)
Hb	N/A	N/A	X	N/A	N/A	N/A
Anemia	↑	aHR 5.547 (1.235–24.916)	N/A	N/A	↑	aHR 1.852 (1.265–2.712)
Platelet	↑	aOR 1.090 (1.008–1.178)	N/A	N/A	N/A	N/A
Thrombocytopenia	N/A	N/A	N/A	N/A	N/A	N/A
CRP	↑	aHR 1.220 (1.058–1.407)	X	N/A	N/A	N/A
High CRP	↑	aHR 8.960 (1.657–48.462)	N/A	N/A	↑	aHR 1.520 (1.081–2.137)
ESR	↑	aHR 1.020 (1.016–1.024)	N/A	N/A	X	N/A
High ESR	↑	aHR 1.849 (1.140–2.999)	N/A	N/A	N/A	N/A
Albumin	N/A	N/A	N/A	N/A	N/A	N/A
Hypoalbuminemia	↑	aHR 3.770 (2.697–5.270)	N/A	N/A	N/A	N/A

AFB, acid-fast bacillus; aHR, adjusted hazard ratio; aOR, adjusted odds ratio; CRP, C-reactive protein; ESR, erythrocyte sedimentation rate; Hb, hemoglobin; HIV, human immunodeficiency virus; M. avium, *Mycobacterium avium*; M. abscessus, *Mycobacterium abscessus*; M. kansasii, *Mycobacterium kansasii*; M. xenopi, *Mycobacterium xenopi*; N/A, not applicable; WBC, white blood cell.

“↑” means the factor was significantly associated with increased risk, while “↓” means the factor was significantly associated with decreased risk. “X: means that the factor was not significantly associated in multivariable analysis. “N/A” means there was no available data or the factor was only analyzed in univariable analysis.

Among the underlying diseases, history of tuberculosis (TB) (adjusted HR = 2.749; 95% CI 1.341–5.637; I^2^ = 0; two studies), diabetes (adjusted HR = 2.062; 95% CI 1.194–3.562; I^2^ = 30.7%; three studies), chronic heart disease (adjusted HR = 1.959; 95% CI 1.093–3.509; I^2^ = 61.3%; two studies), malignancy (adjusted HR = 2.213; 95% CI 1.680–2.914; I^2^ = 11.7%; five studies), and systemic immunosuppression (adjusted HR = 2.126; 95% CI 1.311–3.450; I^2^ = 0; two studies) were significantly associated with increased all-cause mortality in multivariable analysis. In one study, chronic liver disease was significantly associated with increased all-cause mortality (adjusted HR = 1.860; 95% CI 1.242–2.785) after adjusting for covariates^9^.

Among the patients’ symptoms, hemoptysis was significantly associated with decreased all-cause mortality after adjusting covariates in one study (adjusted HR = 0.542; 95% CI 0.414–0.709)^20^.

Among radiologic findings, presence of cavity (adjusted HR = 2.380; 95% CI 1.866–3.037; I^2^ = 0; 10 studies; adjusted OR = 3.176; 95% CI 1.369–7.369; one study) and consolidative pattern (adjusted HR = 4.895; 95% CI 2.997–7.996; I^2^ = 0; two studies) were significantly associated with increased all-cause mortality in multivariable analysis.

Among the laboratory findings, acid-fast bacillus (AFB) smear positivity (adjusted HR = 2.456; 95% CI 1.460–4.130; I^2^ = 0; two studies), increasing erythrocyte sedimentation rate (ESR) (adjusted HR = 1.020; 95% CI 1.016–1.024; I^2^ = 0; two studies), and hypoalbuminemia (adjusted HR = 3.770; 95% CI 2.697–5.270; I^2^ = 0; three studies) were significantly associated with increased all-cause mortality in multivariable analysis. In single studies, anemia (adjusted HR = 5.547; 95% CI 1.235–24.916)^31^, increasing platelet count (adjusted OR = 1.090; 95% CI 1.008–1.178)^11^, increasing C-reactive protein (CRP) (adjusted HR = 1.220; 95% CI 1.058–1.407)^19^, high CRP (adjusted HR = 8.960; 95% CI 1.657–48.462)^28^, and high ESR (adjusted HR = 1.849; 95% CI 1.140–2.999)^20^ were significantly associated with increased all-cause mortality after adjusting for covariates.

After adjusting for covariates, treatment with rifamycin regimen in *M. xenopi* lung disease was significantly associated with decreased all-cause mortality (adjusted HR = 0.330; 95% CI 0.151–0.723) in one study^15^.

### Clinical progressive disease with treatment

**Figure 3:**
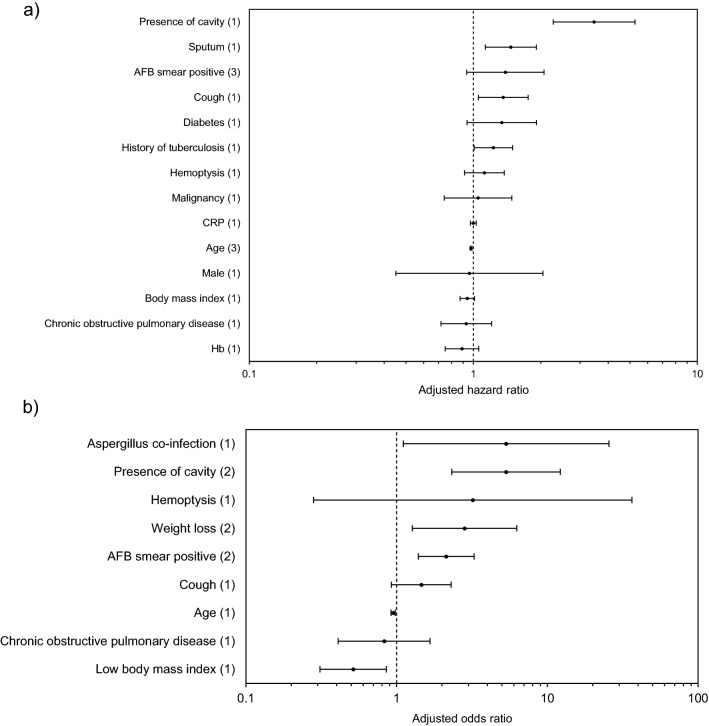
Forest plots displaying the pooled hazard ratios estimating the association between factors and clinical progressive disease with treatment. (**a**) Analysis of adjusted hazard ratios. (**b**) Analysis of adjusted odds ratios. The numbers within the parentheses mean the numbers of studies analyzed. All adjusted risk ratios were estimated in multivariable analysis.

A total of 2,782 patients from 10 studies were included in the review of factors associated with clinical progressive disease with treatment^12,17,34–41^. Figures 3 and S2 present the pooled adjusted RRs and unadjusted RRs, which estimated the association between factors and clinical progressive disease using forest plots. The pooled risk ratios for each factor were presented in Appendix S6.

The overall rate of clinical progressive disease was 46% (95% CI 39%–53%; I^2^ = 91.0%). In baseline demographics, low BMI was significantly associated with decreased clinical progression after adjusting for covariates in one study (adjusted OR = 0.515; 95% CI 0.310–0.855)^36^. Increasing age was significantly associated with decreased clinical progression in multivariable analysis (adjusted HR = 0.976; 95% CI 0.967–0.985; I^2^ = 0; three studies; adjusted OR = 0.950; 95% CI 0.920–0.980; one study).

Among underlying diseases, history of TB (adjusted HR = 1.230; 95% CI 1.009–1.499)^38^ and Aspergillus co-infection (adjusted OR = 5.330; 95% CI 1.107–25.662)^12^ were significantly associated with increased clinical progression after adjusting for covariates in single studies.

Regarding the patient’s symptoms, loss of weight (adjusted OR = 2.822; 95% CI 1.271–6.268; I^2^ = 0; two studies) was significantly associated with increased clinical progression in multivariable analysis. In one study, cough (adjusted HR = 1.360; 95% CI 1.053–1.756) and increased sputum (adjusted HR = 1.470; 95% CI 1.131–1.911) were significantly associated with increased clinical progression after adjusting for covariates^38^.

Among the radiologic findings, presence of cavity (adjusted HR = 3.460; 95% CI 2.273–5.267; one study; adjusted OR = 5.324; 95% CI 2.323–12.199; I^2^ = 0; two studies) was significantly associated with increased clinical progression in multivariable analysis.

Among the laboratory findings, AFB smear positivity (adjusted OR = 2.132; 95% CI 1.393–3.263; I^2^ = 0; two studies) was significantly associated with increased clinical progression in multivariable analysis.

### Radiographic progressive disease

**Figure 4:**
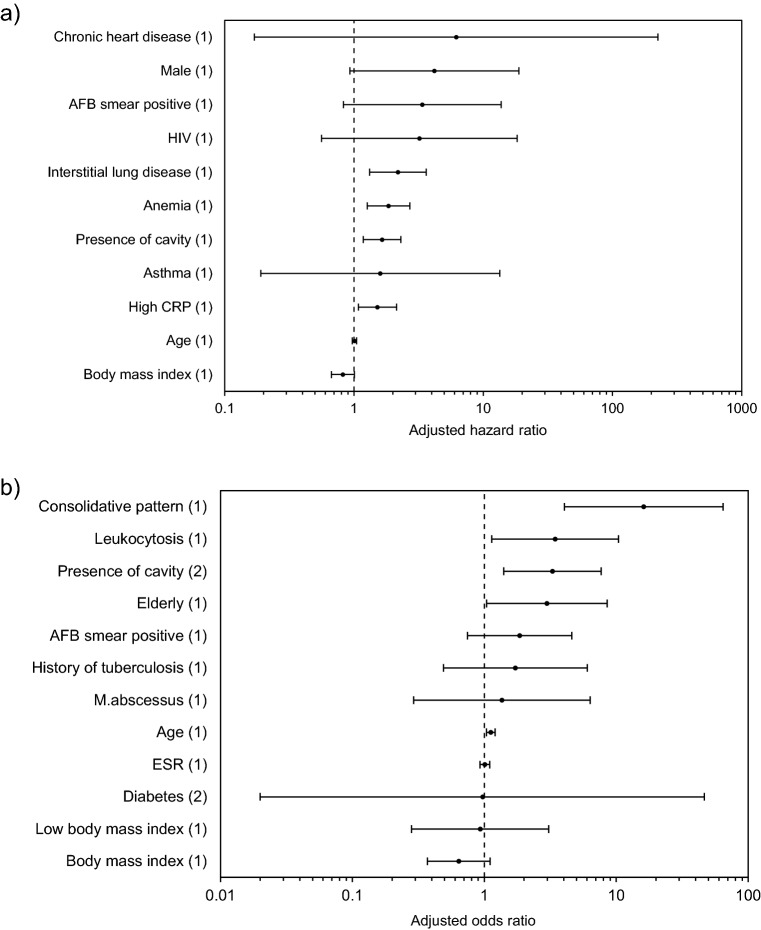
Forest plots displaying the pooled hazard ratios estimating the association between factors and radiographic progressive disease. (**a**) Analysis of adjusted hazard ratios. (**b**) Analysis of adjusted odds ratios. The numbers within the parentheses mean the numbers of studies analyzed. All adjusted risk ratios were estimated in multivariable analysis.

Total 1,439 patients from 11 studies were included in the review of factors associated with radiographic progressive disease^20,30,42–50^. Figures 4 and S3 present the pooled adjusted RRs and unadjusted RRs, which estimated the association between factors and progressive disease using forest plots. The pooled risk ratios for each factor were presented in Appendix S7.

The overall rate of radiographic progressive disease was 43% (95% CI 31%–55%, I^2^ = 95.1%). In single studies, increasing age (adjusted OR = 1.120; 95% CI 1.038–1.209)^49^ and being elderly (adjusted OR = 2.980; 95% CI 1.041–8.534)^48^ was significantly associated with increased radiographic progression after adjusting for covariates.

Interstitial lung disease (adjusted HR = 2.191; 95% CI 1.325–3.623) was significantly associated with increased radiographic progression after adjusting for covariates in one study^20^.

After adjusting for covariates, presence of cavity (adjusted HR = 1.651; 95% CI 1.181–2.308; one study; adjusted OR = 3.283; 95% CI 1.405–7.673; I^2^ = 0; two studies) was significantly associated with increased radiographic progression. Additionally, the consolidative pattern (adjusted OR = 16.150; 95% CI 4.048–64.429) was significantly associated with increased radiographic progression after adjusting for covariates in one study^49^.

Among the laboratory findings, anemia (adjusted HR = 1.852; 95% CI 1.265–2.712)^20^, high CRP (adjusted HR = 1.520; 95% CI 1.081–2.137)^20^, and leukocytosis (adjusted OR = 3.440; 95% CI 1.139–10.390)^48^ were significantly associated with increased radiographic progression after adjusting for covariates in single studies.

### Heterogeneity between studies

Seventeen pooled effect estimates were identified as having substantial heterogeneity (Appendix S8), 10 of which were analyzed for all-cause mortality, four were analyzed for clinical progressive disease with treatment, and three were analyzed for radiographic progressive disease as an outcome.

### Publication bias and certainty of evidence

Publication bias was observed in two estimates (adjusted HR of age and presence of cavity for all-cause mortality) when assessed by Egger’s and Begg’s tests (Appendix S9). After the trim and fill method, the overall effect size remains unchanged. The levels of certainty of evidence regarding associated factors are described in Appendix S10. All associated factors had very low or low levels of evidence certainty.

## Discussion

This systematic review and meta-analysis described the factors associated with all-cause mortality and progressive disease in patients with NTM-LD. After adjusting for covariates, older age, male sex, history of TB, diabetes, chronic heart disease, malignancy, systemic immunosuppression, chronic liver disease, presence of cavity, consolidative radiologic feature, AFB smear positivity, hypoalbuminemia, anemia, increasing platelet count, high CRP, and high ESR were significantly associated with increased all-cause mortality, while increasing BMI, hemoptysis, and treatment with rifamycin regimen (in *M. xenopi*) were significantly associated with decreased all-cause mortality. History of TB, Aspergillus co-infection, cough, increased sputum, weight loss, presence of cavity, and AFB smear positivity were significantly associated with increased clinical progression after adjusting for covariates, which were consistent findings with current guidelines for the treatment of NTM-LD^1,8^. On the other hand, older age, and low BMI were significantly associated with decreased clinical progression. After adjusting for covariates, older age, interstitial lung disease, presence of cavity, consolidative radiologic feature, anemia, high CRP, and leukocytosis were significantly associated with increased radiographic progression. Older age, history of tuberculosis, presence of cavity, consolidative radiologic feature, AFB smear positivity, anemia, and high CRP were common significant factors associated with the all-cause mortality and clinical or radiographic progressive disease of NTM-LD. With our findings, physicians can comprehensively evaluate factors associated with poor prognosis and closely follow up with patients with NTM-LD, especially those that may be at a higher risk. However, the benefit of any treatment regimen for NTM-LD is unclear because of the insufficient number of RCTs. Additionally, the certainty of evidence from our findings was low to very low. Therefore, additional prospective studies are required to identify patients who are at high risk for mortality and progressive disease and may benefit from treatment for NTM-LD.

Among our findings, male sex, and high ESR levels were identified as significant factors associated with all-cause mortality. These findings were consistent with the results of a systematic review of all-cause mortality and prognostic factors for MAC-LD^51^. They were also consistent with components of the BACES score, a new scoring system for predicting all-cause mortality of NTM-LD; it is composed of low BMI, advanced age, presence of cavity, elevated ESR, and male sex^52^. However, they were not significantly associated with progressive disease in our study. These factors are considered confounding variables as they may affect mortality but do not directly affect the NTM-LD progression.

Interestingly, hemoptysis was identified as a significant factor associated with decreased all-cause mortality. However, it is uncertain how hemoptysis and mortality are linked. In general hospitals, NTM-LD patients frequently experience hemoptysis; however, most cases are non-massive or non-life-threatening^53^. A previous study revealed that hemoptysis secondary to NTM-LD occurred more frequently in relatively young patients, and all patients with hemoptysis survived during the observation period^54^. In our study, hemoptysis still exhibited a negative correlation with all-cause mortality, despite the adjustment for age. Therefore, hemoptysis in NTM-LD patients is mostly non-critical and may be related to the early detection of NTM-LD, which can contribute to earlier intervention and a better prognosis.

When evaluating the factors associated with the progressive disease of NTM-LD, it is important to correctly define the progressive disease. Our study demonstrated paradoxical results in terms of age and BMI. Although increasing age was significantly associated with increased all-cause mortality and radiographic progression, it was significantly associated with decreased clinical progression with treatment. Additionally, low BMI was significantly associated with increased all-cause mortality but decreased clinical progression, though low BMI was not significantly associated with radiographic progression. These results may be due to heterogeneous definitions of progressive disease in NTM-LD. Most studies defined progressive disease based on aggravation of clinical symptoms as well as radiographic deterioration. However, the clinical symptoms of NTM-LD are not correlated with the severity of the radiologic features^55^. Clinical symptoms are often exacerbated by other causes than the progression. Additionally, physicians may hesitate the initiation of anti-NTM treatment in the elderly and malnourished patients. It is necessary to establish a standardized definition of progressive disease of NTM-LD and set goals for future research.

This study had several limitations. First, the number of studies involved in deriving each pooled effect estimate was quite small. Therefore, the results could be imprecise, and a publication bias could not be discounted. However, when publication bias was suspected following the Egger's test and Begg's test, the estimates were corrected using the trim and fill method.

Second, the estimates were adjusted for a variable set of adjustment factors, even though most studies adjusted for at least one of age, sex, BMI, or presence of cavity.

Third, meta-regression and subgroup analysis were not performed due to the small number of studies included, although substantial heterogeneity was found in some pooled effect estimates. We considered the differences in the setting of adjustment, follow-up period, countries, NTM species, radiologic features, and the timing among the characteristics of the included studies, as causes of heterogeneity. Especially, factors may have different association according to NTM species or presence of cavity. However, there were no analyses available for association with factors stratified by NTM species or presence of cavity. These factors should be accounted for when estimating pooled effects with substantial heterogeneity.

Lastly, the evaluated certainty of the evidence of all the included studies was very low or low, and most had moderate or high ROBs. Therefore, further well-designed prospective studies are required.

Although there were some limitations, this study is the first systematic review and meta-analysis investigating the factors associated with all-cause mortality, progressive disease in patients with NTM-LD. The significance of this lies in the systematic compilation of factors that are related to outcomes in NTM-LD, which aims to improve the accuracy of underpowered study results. This systematic approach helps to identify important factors that may have been overlooked in previous studies, and by taking these factors into account, future studies may be designed more accurately to evaluate outcomes in NTM-LD.

In conclusion, older age, history of TB, presence of cavity, consolidative radiologic features, AFB smear positivity, anemia, and high CRP were common significant factors associated with the all-cause mortality and clinical or radiographic progressive disease of NTM-LD. These factors may directly affect NTM-LD related mortality. The future prediction models for the prognosis of NTM-LD need to include these factors.

## Methods

### Protocol and registration

This review was conducted following the guidelines for systematic review and meta-analysis of prognostic factor studies, and reported following the Meta-analysis of Observational Studies in Epidemiology and Preferred Reporting Items for Systematic Review and Meta-analysis (PRISMA)^56–58^. The systematic review protocol was registered in the International Prospective Register of Systematic Reviews (PROSPERO, CRD42021246363).

### Eligibility criteria

This systematic review included studies that satisfied the following eligibility criteria: (1) the study subjects were adult patients diagnosed with NTM-LD according to the 2007 American ATS Thoracic Society (ATS)/Infectious Diseases Society of America (IDSA) criteria^1,2^; (2) the factors associated with to all-cause mortality and/or progressive disease were evaluated; and (3) must be a human clinical study; however, case reports, case series, reviews, and guidelines were excluded. We excluded studies comprising NTM-LD patients who did not meet the 2007 ATS/IDSA criteria.

### Information sources and search strategy

The following databases were searched for eligible published and unpublished studies dated between January 1, 2007, to April 12, 2021: Medline, Embase, Cochrane Central Register of Controlled Trials, and Web of Science. The search strategy was designed according to the Peer Review of Electronic Search Strategies^59^. To complement the search strategy, manual searches were performed to find additional references cited in recent articles, systematic reviews, and meta-analyses. There were no restrictions on ethnicity or language in the search strategy. The detailed search strategy is presented in Appendix S11.

### Study selection

Two independent reviewers (HH and HWL) conducted the study selection based on the PRISMA flow diagram^58^. Duplicated studies were removed following the study title and name of the first author. The titles, abstracts, and keywords were screened to identify potentially eligible studies. After screening, a full-text review was conducted to confirm their eligibility by independent reviewers (HH and HWL). If the study population was same and the study period was similar, it was regarded as the same data source. For multiple studies with the same data source, only the study with the largest sample size was selected.

### Data extraction and quality assessment

HH and HWL extracted data on study characteristics and the potentially associated factors. Data related to study characteristics included the first author, publication year, country, study design, study period, inclusion criteria, and study outcomes. The potentially associated factors observed during the review process were patient age, sex, BMI, smoking status, comorbidities, symptoms, radiologic patterns, NTM species, laboratory findings, treatment regimen, and the duration of treatment.

The ROB was assessed by the reviewers using the Quality in Prognostic Studies tool^60^. If there was any uncertainty or disagreement during the study selection process, data extraction, or ROB assessment, a consensus was attained through discussion.

### Outcomes

The primary outcomes were all-cause mortality and progressive disease. Progressive disease was defined as the radiographic deterioration of NTM-LD (radiographic progressive disease) or the initiation of anti-NTM treatment at the discretion of the physician (clinical progressive disease).

### Data synthesis and meta-analysis

The risk ratios (RRs), including hazard ratios (HRs) and odds ratios (ORs), were extracted as effect measures. The RRs were collected and synthesized separately according to the type of RR and whether the estimates were adjusted. If the relationship between a factor and an outcome was given only in the form of a two-by-two table, an unadjusted OR was calculated. The adjusted RRs were preferentially used to interpret the results of the analyses.

Considering the heterogeneity in demographic characteristics and clinical settings, we used the DerSimonian and Laird random-effects model for meta-analysis using the pre-estimated RRs. Additionally, the overall rates of all-cause mortality, clinical progressive disease, and radiographic progression were meta-analyzed using random-effects model. The synthesized effect size was presented with a 95% confidence interval (CI). A forest plot was used to visualize the overall results of the meta-analysis. Heterogeneity among the included studies was evaluated using I^2^ statistics, and substantial heterogeneity was defined as I^2^ ≥ 50%. Subgroup analysis according to the type of progressive disease was performed to evaluate the cause of substantial heterogeneity. Egger’s and Begg’s tests were used to evaluate the publication bias and the trim-and-fill method was used if any bias was suspected. Statistical significance was set at* P* < 0.05. All statistical analyses were performed using Stata 14.2 (StataCorp LLC, College Station, TX, USA).

### Certainty of evidence

The Grading of Recommendations, Assessment, Development, and Evaluations (GRADE) system was used to assess the certainty of evidence^61,62^.

## Supplementary Information

Supplementary Information 1.

Supplementary Information 2.

Supplementary Information 3.

Supplementary Information 4.

Supplementary Information 5.

Supplementary Information 6.

Supplementary Information 7.

Supplementary Information 8.

Supplementary Information 9.

Supplementary Information 10.

Supplementary Information 11.

Supplementary Information 12.

Supplementary Information 13.

Supplementary Information 14.

## Data Availability

The datasets generated during and/or analysed during the current study are available from the corresponding author on reasonable request.

## Abbreviations

AFB: Acid-fast bacillus
ATS: American Thoracic Society
BMI: Body mass index
CRP: C-reactive protein
ESR: Erythrocyte sedimentation rate
GRADE: The Grading of Recommendations, Assessment, Development, and Evaluations
HR: Hazard ratio
IDSA: Infectious Diseases Society of America
MAC: *M. avium* Complex
NTM-LD: Nontuberculous mycobacterial lung disease
OR: Odds ratio
PRISMA: Preferred Reporting Items for Systematic Review and Meta-analysis
RCT: Randomized clinical trial
ROB: Risk of bias
RR: Risk ratio
TB: Tuberculosis
