# Supplementary material for: The factors associated with mortality and progressive disease of nontuberculous mycobacterial lung disease: a systematic review and meta-analysis
Source: Sci Rep. 2023 May 5;13:7348. doi: 10.1038/s41598-023-34576-z (PMC10162985; doi:10.1038/s41598-023-34576-z)
Supplement: Supplementary file 1 — Supplementary Information 1. [file 41598_2023_34576_MOESM1_ESM.docx]

**Appendix S1. Excluded references through the full-text review**

| **No.** | **Study title** | **Author** | **Journal (published year)** | **Major reason for exclusion** |
| --- | --- | --- | --- | --- |
| 1 | BACES Score for Predicting Mortality in Nontuberculous Mycobacterial Pulmonary Disease | Kim, H. J. et al | American Journal of Respiratory and Critical Care Medicine (2020) | This study was eligible for our study, but we used another reference with the same data source. |
| 2 | Change in lung function in never-smokers with nontuberculous mycobacterial lung disease: A retrospective study | Kobayashi, T. et al | Journal of Clinical Tuberculosis and Other Mycobacterial Diseases (2018) | This study has insufficient data for associated factors. |
| 3 | Characteristics associated with progression in patients with of nontuberculous mycobacterial lung disease: a prospective cohort study | Kim, S. J. et al | BMC Pulmonary Medicine (2017) | This study was eligible for our study, but we used another reference with the same data source. |
| 4 | Clinical features and treatment outcomes of Mycobacterium chimaera lung disease and antimicrobial susceptibility of the mycobacterial isolates | Chen, L. C. et al | Journal of Infection (2020) | This study was eligible for our study, but we used another reference with the same data source. |
| 5 | Clinical significance of Aspergillus species isolated from respiratory specimens in patients with Mycobacterium avium complex lung disease | Furuuchi, K. et al | European Journal of Clinical Microbiology & Infectious Diseases (2018) | This study was eligible for our study, but we used another reference with the same data source. |
| 6 | Clinical significance of mycobacterial genotyping in Mycobacterium avium lung disease in Korea | Kim, S. Y. et al | The International Journal of Tuberculosis and Lung Disease (2012) | This study was eligible for our study, but we used another reference with the same data source. |
| 7 | Clinical significance of the radiological severity score in Mycobacterium avium complex lung disease patients | Furuuchi, K. et al | The International Journal of Tuberculosis and Lung Disease (2017) | This study has insufficient data for associated factors. |
| 8 | Down-Regulation of Serum High-Mobility Group Box 1 Protein in Patients with Pulmonary Tuberculosis and Nontuberculous Mycobacterial Lung Disease | Kim, S. Y. et al | Tuberculosis and Respiratory Diseases (2017) | This study has insufficient data for associated factors. |
| 9 | Extensive Lung Resection for Nontuberculous Mycobacterial Lung Disease With Multilobar Lesions | Yamada, K. et al | The Annals of Thoracic Surgery (2021) | This study has insufficient data for associated factors. |
| 10 | Genotyping of Mycobacterium intracellulare isolates and clinical characteristics of lung disease | Kim, S. Y. et al | The International Journal of Tuberculosis and Lung Disease (2013) | This study was eligible for our study, but we used another reference with the same data source. |
| 11 | Impact of different subspecies on disease progression in initially untreated patients with Mycobacterium avium complex lung disease | Pan, S. W. et al | Clinical Microbiology and Infection (2020) | The outcome of study is ineligible for our study. |
| 12 | Latent class analysis to define radiological subgroups in pulmonary nontuberculous mycobacterial disease | Cowman, S. A. et al | BMC Pulmonary Medicine (2018) | This study has insufficient data for associated factors. |
| 13 | Long-term radiographic outcome of nodular bronchiectatic Mycobacterium avium complex pulmonary disease | Kitada, S. et al | The International Journal of Tuberculosis and Lung Disease (2012) | The study population did not meet the eligibility criteria of our study. |
| 14 | Long-Term Treatment Outcome of Progressive Mycobacterium Avium Complex Pulmonary Disease | Fukushima, K. et al | Journal of Clinical Medicine (2020) | The outcome of study is ineligible for our study. |
| 15 | Long-term, low-dose erythromycin monotherapy for Mycobacterium avium complex lung disease: A propensity score analysis | Komiya, K. et al | International Journal of Antimicrobial Agents (2014) | The outcome of study is ineligible for our study. |
| 16 | Microbiological Persistence in Patients With Mycobacterium avium Complex Lung Disease: The Predictors and the Impact on Radiographic Progression | Pan, S. W. et al | Clinical Infectious Diseases (2017) | This study was eligible for our study, but we used another reference with the same data source. |
| 17 | Mycobacterial genotypes are associated with clinical manifestation and progression of lung disease caused by Mycobacterium abscessus and Mycobacterium massiliense | Shin, S. J. et al | Clinical Infectious Diseases (2013) | This study was eligible for our study, but we used another reference with the same data source. |
| 18 | Nodular bronchiectatic Mycobacterium avium complex pulmonary disease. Natural course on serial computed tomographic scans | Lee, G. et al | Annals of the American Thoracic Society (2013) | This study was eligible for our study, but we used another reference with the same data source. |
| 19 | Non-tuberculous slow-growing mycobacterial pulmonary infections in non-HIV-infected patients in south London | Davies, B. S. et al | Scandinavian Journal of Infectious Diseases (2012) | This study has insufficient data for associated factors. |
| 20 | Predictors of radiographic progression for NTM-pulmonary disease diagnosed by bronchoscopy | Huang, H. L. et al | Respiratory Medicine (2020) | This study was eligible for our study, but we used another reference with the same data source |
| 21 | Prognostic factors and radiographic outcomes of nontuberculous mycobacterial lung disease in rheumatoid arthritis | Yamakawa, H. et al | The Journal of Rheumatology (2013) | This study was eligible for our study, but we used another reference with the same data source. |
| 22 | Prognostic factors of 634 HIV-negative patients with Mycobacterium avium complex lung disease | Hayashi, M. et al | American Journal of Respiratory and Critical Care Medicine (2012) | This study was eligible for our study, but we used another reference with the same data source. |
| 23 | Progression and Treatment Outcomes of Lung Disease Caused by Mycobacterium abscessus and Mycobacterium massiliense | Park, J. et al | Clinical Infectious Diseases (2017) | This study was eligible for our study, but we used another reference with the same data source. |
| 24 | Quantitative assessment of erector spinae muscles in patients with Mycobacterium avium complex lung disease | Asakura, T. et al | Respiratory Medicine (2018) | This study was eligible for our study, but we used another reference with the same data source. |
| 25 | The Clinical Significance of Programmed Death-1, Regulatory T Cells and Myeloid Derived Suppressor Cells in Patients with Nontuberculous Mycobacteria-Lung Disease | Shu, C. C. et al | Journal of Clinical Medicine (2019) | This study was eligible for our study, but we used another reference with the same data source. |
| 26 | The impact of adjuvant surgical treatment of nontuberculous mycobacterial pulmonary disease on prognosis and outcome | Fukushima, K. et al | Respiratory Research (2020) | This study has insufficient data for associated factors. |
| 27 | The impact of different antibiotic treatment regimens on mortality in Mycobacterium avium complex pulmonary disease (MAC-PD): A population-based cohort study | Sarah, K. Brode et al | European Respiratory Journal (2020) | This study has insufficient data for associated factors. |
| 28 | The impact of different antibiotic treatment regimens on mortality in Mycobacterium avium complex pulmonary disease: a population-based cohort study | Brode, S. K. et al | European Respiratory Journal (2020) | This study has insufficient data for associated factors. |
| 29 | The impact of low subcutaneous fat in patients with nontuberculous mycobacterial lung disease | Lee, S. J. et al | Lung (2014) | This study has insufficient data for associated factors. |
| 30 | The microbiological and clinical effects of combined therapy according to guidelines on the treatment of pulmonary Mycobacterium avium complex disease in Japan - including a follow-up study | Kobashi, Y. et al | Respiration (2007) | The outcome of study is ineligible for our study. |
| 31 | Unilateral Lung Involvement of Nodular Bronchiectatic Mycobacterium Avium Complex Pulmonary Diseases: Proportion and Evolution on Serial CT Studies | Choi, Y. et al | American Journal of Roentgenology (2019) | This study was eligible for our study, but we used another reference with the same data source. |
| 32 | Use of soluble triggering receptor expressed on myeloid cells-1 in non-tuberculous mycobacterial lung disease | Shu, C. C. et al | The International Journal of Tuberculosis and Lung Disease (2011) | This study has insufficient data for associated factors. |
