# Supplementary material for: The factors associated with mortality and progressive disease of nontuberculous mycobacterial lung disease: a systematic review and meta-analysis
Source: Sci Rep. 2023 May 5;13:7348. doi: 10.1038/s41598-023-34576-z (PMC10162985; doi:10.1038/s41598-023-34576-z)
Supplement: Supplementary file 2 — Supplementary Information 2. [file 41598_2023_34576_MOESM2_ESM.docx]

**Appendix S2. Assessment of the risk of bias**

| Study | Study participation | Study attrition | Prognostic factor measurement | Outcome  measurement | Adjustment for  other prognositic factors | Statistical analysis and reporting | Overall risk bias |
| --- | --- | --- | --- | --- | --- | --- | --- |
| Abate, G. (2021) | moderate | high | moderate | low | moderate | moderate | moderate |
| Akahori, D. (2019) | moderate | high | moderate | low | moderate | moderate | moderate |
| Andréjak, C. (2009) | low | moderate | moderate | low | high | high | moderate |
| Asakura, T. (2017) | moderate | high | low | low | moderate | low | moderate |
| Asakura, T. (2020) | moderate | high | low | moderate | moderate | low | moderate |
| Chang, C. L. (2020) | low | high | low | moderate | moderate | low | moderate |
| Fleshner, M. (2016) | moderate | high | high | low | moderate | moderate | high |
| Fukushima, K. (2021) | high | low | low | low | moderate | moderate | moderate |
| Gochi, M. (2015) | low | high | low | low | low | low | low |
| Hachisu, Y. (2020) | moderate | high | moderate | low | moderate | moderate | moderate |
| Hong, J. Y. (2016) | high | low | moderate | moderate | moderate | low | moderate |
| Hwang, J. A. (2017) | moderate | high | moderate | low | moderate | low | moderate |
| Inomata, T. (2018) | moderate | high | low | low | high | moderate | moderate |
| Ito, Y. (2012) | high | high | low | low | moderate | high | high |
| Jenkins, P. A. (2008) | low | low | low | low | high | low | moderate |
| Jhun, B. W. (2020) | low | moderate | moderate | low | low | low | low |
| Kadota, T. (2016) | moderate | low | low | moderate | moderate | moderate | moderate |
| Kang, H. R. (2021) | moderate | moderate | low | low | moderate | low | moderate |
| Kikuchi, T. (2009) | high | low | low | moderate | moderate | moderate | moderate |
| Kim, H. J. (2019) | low | low | low | low | moderate | low | low |
| Kim, S. J. (2014) | high | high | low | moderate | low | moderate | moderate |
| Kodaka, N. (2020) | high | high | moderate | moderate | moderate | moderate | high |
| Kumagai, S. (2017) | moderate | high | moderate | low | moderate | low | moderate |
| Kwon, B. S. (2019) | high | moderate | low | moderate | moderate | low | moderate |
| Liu, C. J. (2019) | high | moderate | moderate | moderate | moderate | moderate | high |
| Matsuda, S. (2020) | moderate | high | moderate | moderate | moderate | low | moderate |
| Moon, S. M. (2019) | high | high | low | moderate | low | low | moderate |
| Moon, S. W. (2020) | moderate | high | low | low | moderate | low | moderate |
| Mori, S. (2020) | moderate | high | moderate | low | moderate | low | moderate |
| Moriyama, M. (2016) | low | low | high | moderate | high | moderate | moderate |
| Naito, M. (2018) | moderate | high | low | low | low | low | moderate |
| Ogawa, T. (2021) | high | high | low | low | moderate | low | moderate |
| Oshitani, Y. (2021) | high | high | moderate | moderate | moderate | moderate | high |
| Provoost, J. (2018) | high | moderate | low | moderate | moderate | low | moderate |
| Raats, D. (2021) | moderate | high | moderate | low | moderate | low | moderate |
| Rawson, T. M. (2016) | high | moderate | moderate | moderate | low | low | moderate |
| Shirai, T. (2020) | high | moderate | low | low | moderate | low | moderate |
| Shu, C. C. (2011) | high | high | moderate | low | moderate | high | high |
| Ushiki, A. (2011) | high | high | low | moderate | moderate | moderate | high |
| Wang, P. H. (2020) | moderate | high | low | low | moderate | low | moderate |
| Yamamoto, Y. (2021) | moderate | high | moderate | moderate | moderate | low | moderate |
|  |  |  |  |  |  |  |  |
|  |  |  |  |  |  |  |  |
|  |  |  |  | **Risk of bias ratings** | **low** | **moderate** | **high** |
|  |  |  |  |  |  |  |  |
