# Supplementary material for: The factors associated with mortality and progressive disease of nontuberculous mycobacterial lung disease: a systematic review and meta-analysis
Source: Sci Rep. 2023 May 5;13:7348. doi: 10.1038/s41598-023-34576-z (PMC10162985; doi:10.1038/s41598-023-34576-z)
Supplement: Supplementary file 4 — Supplementary Information 4. [file 41598_2023_34576_MOESM4_ESM.docx]

**Appendix S4. Factors significantly associated with all-cause mortality and progressive disease in univariable analysis**

| **Outcome: all-cause mortality** | | | |
| --- | --- | --- | --- |
| **Factors** | **Estimated effect size (95% confidence interval)** | **Number of included studies** | **I^2^ for heterogeneity** |
| Male | uHR 1.847 (1.078-3.164) | 3 | 14.1% |
| Male | uOR 2.287 (1.379-3.794) | 3 | 0 |
| Body mass index | uHR 0.876 (0.799-0.959) | 2 | 0 |
| Ever-smoking | uHR 2.666 (2.111-3.369) | 4 | 8.2% |
| Ever-smoking | uOR 3.206 (1.140-9.014) | 1 | - |
| Diabetes | uHR 2.471 (1.863-3.278) | 2 | 0 |
| Chronic lung disease | uHR 3.703 (2.490-5.508) | 2 | 0 |
| Chronic obstructive pulmonary disease | uOR 2.137 (1.194-3.823) | 3 | 0 |
| Bronchiectasis | uHR 0.300 (0.090-0.995) | 1 | - |
| Bronchiectasis | uOR 0.379 (0.147-0.977) | 1 | - |
| Asthma | uOR 20.200 (3.366-121.216) | 1 | - |
| Chronic kidney disease | uOR 5.963 (1.613-22.042) | 1 | - |
| Hemoptysis | uHR 0.714 (0.542-0.940) | 2 | 0 |
| Sputum | uHR 1.490 (1.097-2.024) | 1 | - |
| Weight loss | uOR 3.433 (1.587-7.425) | 1 | - |
| Nodular-bronchiectatic pattern | uOR 0.432 (0.216-0.862) | 2 | 0 |
| AFB smear positivity | uHR 1.719 (1.030-2.869) | 4 | 75.7% |
| Hb | uHR 0.778 (0.629-0.962) | 1 | - |
| Anemia | uHR 2.320 (1.377-3.909) | 2 | 0 |
| CRP | uHR 1.560 (1.321-1.843) | 1 | - |
| High CRP | uHR 3.614 (2.296-5.688) | 2 | 23.6% |
| Albumin | uHR 0.286 (0.172-0.474) | 1 | - |
| Treatment duration | uOR 0.917 (0.864-0.973) | 1 | - |
| **Outcome: Clinical progressive disease with treatment** | | | |
| Body mass index | uOR 0.890 (0.795-0.996) | 1 | - |
| Low body mass index | uOR 4.250 (1.184-15.253) | 1 | - |
| Chronic obstructive pulmonary disease | uOR 3.448 (1.906-6.238) | 2 | 0 |
| Hemoptysis | uOR 0.691 (0.486-0.983) | 2 | 0 |
| Mycobacterium kansasii | uOR 3.110 (1.030-9.390) | 1 | - |
| AFB smear positivity | uOR 2.078 (1.148-3.762) | 2 | 0 |
| CRP | uOR 2.700 (1.179-6.184) | 1 | - |
| **Outcome: Radiographic progressive disease** | | | |
| Body mass index | uOR 0.896 (0.815-0.986) | 2 | 0 |
| Any comorbidity | uOR 0.339 (0.141-0.815) | 1 | - |
| Presence of cavity | uOR 2.655 (1.209-5.831) | 2 | 0 |
| Hypoalbuminemia | uOR 2.898 (1.143-7.347) | 2 | 0 |

Abbreviations: AFB, acid-fast bacillus; aHR, adjusted hazard ratio; aOR, adjusted odds ratio; CRP, C-reactive protein; Hb, hemoglobin; uHR, unadjusted hazard ratio; uOR, unadjusted odds ratio
