# Supplementary material for: The factors associated with mortality and progressive disease of nontuberculous mycobacterial lung disease: a systematic review and meta-analysis
Source: Sci Rep. 2023 May 5;13:7348. doi: 10.1038/s41598-023-34576-z (PMC10162985; doi:10.1038/s41598-023-34576-z)
Supplement: Supplementary file 5 — Supplementary Information 5. [file 41598_2023_34576_MOESM5_ESM.docx]

**Appendix S5. The definition of categorical variables in each study**

| Study | Elderly | Low BMI | Chronic lung disease | Interstitial lung disease | Chronic heart disease | Chronic liver disease | Chronic kidney disease | Malignancy | Systemic immunosuppression | Leukocytosis | Anemia | Thrombocytopenia | High CRP | High ESR | Hypoalbuminemia |
| --- | --- | --- | --- | --- | --- | --- | --- | --- | --- | --- | --- | --- | --- | --- | --- |
| Abate, G. | – | – | – | Interstitial lung disease | Congestive heart failure | Chronic liver disease | Chronic kidney disease | Lung cancer | Autoimmune diseases/connective tissue disorder | – | – | – | – | – | – |
| Akahori, D. | – | BMI < 18.5 kg/m^2^ | – | – | – | – | – | – | – | – | – | – | – | – | – |
| Andréjak, C. | – | – | – | – | – | – | – | – | Systemic immunosuppression | – | – | – | – | – | – |
| Asakura, T. (2017) | – | – | – | – | – | – | – | – | – | – | – | – | – | – | – |
| Asakura, T. (2021) | – | – | Underlying pulmonary diseases | – | – | – | – | – | – | – | – | – | – | – | – |
| Chang, C. L. | – | – | – | – | Coronary artery disease | – | – | Lung cancer | Immunosuppressants | – | – | – | – | – | – |
| Fleshner, M. | – | – | – | – | – | – | – | – | – | – | – | – | – | – | – |
| Fukushima, K. | – | – | – | – | – | – | – | – | – | – | – | – | – | – | – |
| Gochi, M. | Age ≥ 70 years | BMI < 18.5 kg/m^2^ | – | Underlying IPF | – | – | – | – | – | – | Hb < 11.3 g/dL | – | CRP ≥ 1.0 mg/dL | ESR ≥40 mm/h | Albumin < 3.5 g/dL |
| Hachisu, Y. | – | – | – | Interstitial lung disease | – | – | – | Tumor past or concurrent | – | – | – | – | – | – | – |
| Hong, J. Y. | Age > 70 years | BMI < 19.5 kg/m^2^ | – | – | – | – | – | – | – | – | – | – | – | – | – |
| Hwang, J. A. | – | – | – | – | – | – | – | – | – | – | – | – | – | – | – |
| Inomata, T. | – | – | – | – | – | – | – | – | – | – | – | – | – | – | – |
| Ito, Y. | Age ≥ 65 years | – | – | Interstitial lung disease | – | – | – | Malignancy | – | – | – | – | – | – | – |
| Jenkins, P. A. et al | – | – | – | – | – | – | – | – | – | – | – | – | – | – | – |
| Jhun, B. W. | Age ≥ 65 years | BMI < 18.5 kg/m^2^ | – | – | Chronic heart disease | Chronic liver disease | – | Pulmonary malignancy | – | – | – | – | – | – | – |
| Kadota, T. | Age ≥ 75 years | – | – | – | – | – | – | – | – | – | – | – | CRP ≥1.0 mg/dL | – | Albumin < 3.5 g/dL |
| Kang, H. R. | – | – | – | – | – | – | – | Malignancy | – | – | – | – | – | – | – |
| Kikuchi, T. | – | – | History of pulmonary disease | – | – | – | – | – | – | – | – | – | – | – | – |
| Kim, H. J. | – | – | – | – | – | – | – | – | – | – | – | – | – | – | – |
| Kim, S. J. | – | – | – | – | – | – | – | Malignancy | – | – | – | – | – | – | – |
| Kodaka, N. | – | – | – | – | – | – | – | – | – | – | – | – | – | – | – |
| Kumagai, S. | Age ≥ 70 years | BMI < 18.5 kg/m^2^ | Respiratory diseases | – | – | – | Chronic renal diseases | Malignancy | – | – | Hb < 10.0 g/dL | – | CRP ≥1.0 mg/dL | – | Albumin < 3.5 g/dL |
| Kwon, B. S. | Age > 60 years | BMI ≤ 18.5 kg/m^2^ | – | Interstitial lung disease | Chronic heart disease | Chronic liver disease | Chronic kidney disease | Malignancy | Use of immunosuppressant | – | – | – | – | – | – |
| Liu, C. J. | Age > 65 years | – | – | Interstitial lung disease | Congestive heart failure | Liver cirrhosis | Chronic kidney disease, stage 3–5 | Lung cancer | Steroid user | Leucocyte >9000/μL | Hb < 12 g/dL | Platelet count < 140 K/uL | CRP ≥ 1.0 mg/dL | – | Albumin < 3.5 g/dL |
| Matsuda, S. | – | – | Underlying pulmonary diseases | – | – | – | – | – | Immunosuppressive therapy | – | – | – | – | – | – |
| Moon, S. M. | – | – | – | – | – | – | – | Malignancy | – | – | – | – | – | – | – |
| Moon, S. W. | Age ≥ 65 years | – | – | – | Cardiovascular disease | Chronic liver disease | Chronic kidney disease | Cancer | – | – | – | – | – | – | – |
| Mori, S. | – | – | – | – | – | – | – | – | – | – | – | – | – | – | – |
| Moriyama, M. | – | – | – | – | – | – | – | – | – | – | – | – | – | – | – |
| Naito, M. | Age ≥ 70 years | – | – | – | – | – | – | – | Use of systemic corticosteroids | – | Hb < 12 g/dL | – | CRP ≥ 5 mg/dL | – | – |
| Ogawa, T. | – | – | – | – | – | – | – | – | – | – | – | – | – | – | – |
| Oshitani, Y. | – | – | – | Interstitial pneumonia | Chronic heart disease | Chronic liver disease | – | – | Steroid and/or immunosuppressive agent use | – | – | – | – | – | – |
| Provoost, J. | – | BMI < 18kg/m^2^ | – | Chronic interstitial lung disease | Chronic heart failure | – | – | History of cured malignant solid tumor | – | – | – | – | – | – | – |
| Raats, D. | – | – | – | – | – | – | – | – | – | – | – | – | – | – | – |
| Rawson, T. M. | Age > 65 years | – | – | – | – | – | – | Cancer ± Chemotherapy | Immunosuppressive therapy | – | – | – | – | – | – |
| Shirai, T. | Age ≥ 70 years | – | Respiratory disease | – | – | – | – | – | – | – | Hb < 10.0 g/dL | – | CRP ≥ 1.0 mg/dL | – | Albumin < 3.5 g/dL |
| Shu, C. C. | – | – | – | – | – | – | – | – | – | – | – | – | – | – | – |
| Ushiki, A. | – | – | – | – | – | – | – | – | – | – | – | – | – | – | – |
| Wang, P. H. | – | – | – | – | – | Cirrhosis | – | Cancer | Autoimmune diseases | – | – | – | – | – | – |
| Yamamoto, Y. | – | – | – | – | – | – | – | – | – | – | – | – | – | – | – |

Abbreviations: BMI, body mass index; CRP, C-reactive protein; ESR, erythrocyte sedimentation rate; Hb, hemoglobin; IPF, idiopathic pulmonary fibrosis
