# Supplementary material for: The factors associated with mortality and progressive disease of nontuberculous mycobacterial lung disease: a systematic review and meta-analysis
Source: Sci Rep. 2023 May 5;13:7348. doi: 10.1038/s41598-023-34576-z (PMC10162985; doi:10.1038/s41598-023-34576-z)
Supplement: Supplementary file 6 — Supplementary Information 6. [file 41598_2023_34576_MOESM6_ESM.docx]

**Appendix S6. Forest plot displaying results of meta-analysis by each factor associated with clinical progressive disease with treatment**

1. **Age**

| **** | **None** |
| --- | --- |
| **Adjusted hazard ratio** | **Unadjusted hazard ratio** |
| **** | **None** |
| **Adjusted odds ratio** | **Unadjusted odds ratio** |

1. **Elderly**

| **None** | **None** |
| --- | --- |
| **Adjusted hazard ratio** | **Unadjusted hazard ratio** |
| **None** | **** |
| **Adjusted odds ratio** | **Unadjusted odds ratio** |

1. **Male**

| **** | **None** |
| --- | --- |
| **Adjusted hazard ratio** | **Unadjusted hazard ratio** |
| **None** | **** |
| **Adjusted odds ratio** | **Unadjusted odds ratio** |

1. **Body mass index**

| **** | **None** |
| --- | --- |
| **Adjusted hazard ratio** | **Unadjusted hazard ratio** |
| **None** | **** |
| **Adjusted odds ratio** | **Unadjusted odds ratio** |

1. **Low body mass index**

| **None** | **None** |
| --- | --- |
| **Adjusted hazard ratio** | **Unadjusted hazard ratio** |
| **** | **** |
| **Adjusted odds ratio** | **Unadjusted odds ratio** |

1. **Ever-smoking**

| **None** | **None** |
| --- | --- |
| **Adjusted hazard ratio** | **Unadjusted hazard ratio** |
| **None** | **** |
| **Adjusted odds ratio** | **Unadjusted odds ratio** |

1. **Any comorbidity**

| **None** | **None** |
| --- | --- |
| **Adjusted hazard ratio** | **Unadjusted hazard ratio** |
| **None** | **** |
| **Adjusted odds ratio** | **Unadjusted odds ratio** |

1. **Diabetes**

| **** | **None** |
| --- | --- |
| **Adjusted hazard ratio** | **Unadjusted hazard ratio** |
| **None** | **** |
| **Adjusted odds ratio** | **Unadjusted odds ratio** |

1. **Chronic lung disease**

| **None** | **** |
| --- | --- |
| **Adjusted hazard ratio** | **Unadjusted hazard ratio** |
| **None** | **** |
| **Adjusted odds ratio** | **Unadjusted odds ratio** |

1. **Chronic obstructive pulmonary disease**

| **** | **None** |
| --- | --- |
| **Adjusted hazard ratio** | **Unadjusted hazard ratio** |
| **** | **** |
| **Adjusted odds ratio** | **Unadjusted odds ratio** |

1. **History of tuberculosis**

| **** | **None** |
| --- | --- |
| **Adjusted hazard ratio** | **Unadjusted hazard ratio** |
| **None** | **** |
| **Adjusted odds ratio** | **Unadjusted odds ratio** |

1. **Bronchiectasis**

| **None** | **None** |
| --- | --- |
| **Adjusted hazard ratio** | **Unadjusted hazard ratio** |
| **None** | **** |
| **Adjusted odds ratio** | **Unadjusted odds ratio** |

1. **Asthma**

| **None** | **None** |
| --- | --- |
| **Adjusted hazard ratio** | **Unadjusted hazard ratio** |
| **None** | **None** |
| **Adjusted odds ratio** | **Unadjusted odds ratio** |

1. **Interstitial lung disease**

| **None** | **None** |
| --- | --- |
| **Adjusted hazard ratio** | **Unadjusted hazard ratio** |
| **None** | **** |
| **Adjusted odds ratio** | **Unadjusted odds ratio** |

1. **Pulmonary hypertension**

| **None** | **None** |
| --- | --- |
| **Adjusted hazard ratio** | **Unadjusted hazard ratio** |
| **None** | **None** |
| **Adjusted odds ratio** | **Unadjusted odds ratio** |

1. **Chronic heart disease**

| **None** | **None** |
| --- | --- |
| **Adjusted hazard ratio** | **Unadjusted hazard ratio** |
| **None** | **** |
| **Adjusted odds ratio** | **Unadjusted odds ratio** |

1. **Chronic liver disease**

| **None** | **None** |
| --- | --- |
| **Adjusted hazard ratio** | **Unadjusted hazard ratio** |
| **None** | **** |
| **Adjusted odds ratio** | **Unadjusted odds ratio** |

1. **Chronic kidney disease**

| **None** | **None** |
| --- | --- |
| **Adjusted hazard ratio** | **Unadjusted hazard ratio** |
| **None** | **** |
| **Adjusted odds ratio** | **Unadjusted odds ratio** |

1. **Malignancy**

| **** | **None** |
| --- | --- |
| **Adjusted hazard ratio** | **Unadjusted hazard ratio** |
| **None** | **** |
| **Adjusted odds ratio** | **Unadjusted odds ratio** |

1. **HIV**

| **None** | **None** |
| --- | --- |
| **Adjusted hazard ratio** | **Unadjusted hazard ratio** |
| **None** | **None** |
| **Adjusted odds ratio** | **Unadjusted odds ratio** |

1. **Systemic immunosuppression**

| **None** | **** |
| --- | --- |
| **Adjusted hazard ratio** | **Unadjusted hazard ratio** |
| **None** | **** |
| **Adjusted odds ratio** | **Unadjusted odds ratio** |

1. **Aspergillus co-infection**

| **None** | **None** |
| --- | --- |
| **Adjusted hazard ratio** | **Unadjusted hazard ratio** |
| **** | **None** |
| **Adjusted odds ratio** | **Unadjusted odds ratio** |

1. **Hemoptysis**

| **** | **None** |
| --- | --- |
| **Adjusted hazard ratio** | **Unadjusted hazard ratio** |
| **** | **** |
| **Adjusted odds ratio** | **Unadjusted odds ratio** |

1. **Cough**

| **** | **None** |
| --- | --- |
| **Adjusted hazard ratio** | **Unadjusted hazard ratio** |
| **** | **** |
| **Adjusted odds ratio** | **Unadjusted odds ratio** |

1. **Sputum**

| **** | **None** |
| --- | --- |
| **Adjusted hazard ratio** | **Unadjusted hazard ratio** |
| **None** | **** |
| **Adjusted odds ratio** | **Unadjusted odds ratio** |

1. **Fatigue**

| **None** | **None** |
| --- | --- |
| **Adjusted hazard ratio** | **Unadjusted hazard ratio** |
| **None** | **** |
| **Adjusted odds ratio** | **Unadjusted odds ratio** |

1. **Dyspnea**

| **None** | **None** |
| --- | --- |
| **Adjusted hazard ratio** | **Unadjusted hazard ratio** |
| **None** | **** |
| **Adjusted odds ratio** | **Unadjusted odds ratio** |

1. **Fever**

| **None** | **None** |
| --- | --- |
| **Adjusted hazard ratio** | **Unadjusted hazard ratio** |
| **None** | **** |
| **Adjusted odds ratio** | **Unadjusted odds ratio** |

1. **Chest pain**

| **None** | **None** |
| --- | --- |
| **Adjusted hazard ratio** | **Unadjusted hazard ratio** |
| **None** | **None** |
| **Adjusted odds ratio** | **Unadjusted odds ratio** |

1. **Weight loss**

| **None** | **None** |
| --- | --- |
| **Adjusted hazard ratio** | **Unadjusted hazard ratio** |
| **** | **None** |
| **Adjusted odds ratio** | **Unadjusted odds ratio** |

1. **Nodular pattern**

| **None** | **None** |
| --- | --- |
| **Adjusted hazard ratio** | **Unadjusted hazard ratio** |
| **None** | **** |
| **Adjusted odds ratio** | **Unadjusted odds ratio** |

1. **Bronchiectatic pattern**

| **None** | **None** |
| --- | --- |
| **Adjusted hazard ratio** | **Unadjusted hazard ratio** |
| **None** | **** |
| **Adjusted odds ratio** | **Unadjusted odds ratio** |

1. **Nodular bronchiectatic pattern**

| **None** | **None** |
| --- | --- |
| **Adjusted hazard ratio** | **Unadjusted hazard ratio** |
| **None** | **** |
| **Adjusted odds ratio** | **Unadjusted odds ratio** |

1. **Presence of cavity**

| **** | **None** |
| --- | --- |
| **Adjusted hazard ratio** | **Unadjusted hazard ratio** |
| **** | **** |
| **Adjusted odds ratio** | **Unadjusted odds ratio** |

1. **Consolidative pattern**

| **None** | **None** |
| --- | --- |
| **Adjusted hazard ratio** | **Unadjusted hazard ratio** |
| **None** | **None** |
| **Adjusted odds ratio** | **Unadjusted odds ratio** |

1. **M. *avium* complex**

| **None** | **None** |
| --- | --- |
| **Adjusted hazard ratio** | **Unadjusted hazard ratio** |
| **None** | **** |
| **Adjusted odds ratio** | **Unadjusted odds ratio** |

1. **M. *abscessus***

| **None** | **None** |
| --- | --- |
| **Adjusted hazard ratio** | **Unadjusted hazard ratio** |
| **None** | **** |
| **Adjusted odds ratio** | **Unadjusted odds ratio** |

1. **M. *kansasii***

| **None** | **None** |
| --- | --- |
| **Adjusted hazard ratio** | **Unadjusted hazard ratio** |
| **None** | **** |
| **Adjusted odds ratio** | **Unadjusted odds ratio** |

1. **M. *xenopi***

| **None** | **None** |
| --- | --- |
| **Adjusted hazard ratio** | **Unadjusted hazard ratio** |
| **None** | **** |
| **Adjusted odds ratio** | **Unadjusted odds ratio** |

1. **AFB smear positive**

| **** | **None** |
| --- | --- |
| **Adjusted hazard ratio** | **Unadjusted hazard ratio** |
| **** | **** |
| **Adjusted odds ratio** | **Unadjusted odds ratio** |

1. **Rifamycin regimen**

| **None** | **None** |
| --- | --- |
| **Adjusted hazard ratio** | **Unadjusted hazard ratio** |
| **None** | **None** |
| **Adjusted odds ratio** | **Unadjusted odds ratio** |

1. **Macrolide regimen**

| **None** | **None** |
| --- | --- |
| **Adjusted hazard ratio** | **Unadjusted hazard ratio** |
| **None** | **None** |
| **Adjusted odds ratio** | **Unadjusted odds ratio** |

1. **Treatment duration**

| **None** | **None** |
| --- | --- |
| **Adjusted hazard ratio** | **Unadjusted hazard ratio** |
| **None** | **None** |
| **Adjusted odds ratio** | **Unadjusted odds ratio** |

1. **Treatment with 3 or more antibiotics**

| **None** | **None** |
| --- | --- |
| **Adjusted hazard ratio** | **Unadjusted hazard ratio** |
| **None** | **None** |
| **Adjusted odds ratio** | **Unadjusted odds ratio** |

1. **WBC**

| **None** | **None** |
| --- | --- |
| **Adjusted hazard ratio** | **Unadjusted hazard ratio** |
| **None** | **** |
| **Adjusted odds ratio** | **Unadjusted odds ratio** |

1. **Leukocytosis**

| **None** | **None** |
| --- | --- |
| **Adjusted hazard ratio** | **Unadjusted hazard ratio** |
| **None** | **None** |
| **Adjusted odds ratio** | **Unadjusted odds ratio** |

1. **Hb**

| **** | **None** |
| --- | --- |
| **Adjusted hazard ratio** | **Unadjusted hazard ratio** |
| **None** | **None** |
| **Adjusted odds ratio** | **Unadjusted odds ratio** |

1. **Anemia**

| **None** | **None** |
| --- | --- |
| **Adjusted hazard ratio** | **Unadjusted hazard ratio** |
| **None** | **None** |
| **Adjusted odds ratio** | **Unadjusted odds ratio** |

1. **Platelet**

| **None** | **None** |
| --- | --- |
| **Adjusted hazard ratio** | **Unadjusted hazard ratio** |
| **None** | **None** |
| **Adjusted odds ratio** | **Unadjusted odds ratio** |

1. **Thrombocytopenia**

| **None** | **None** |
| --- | --- |
| **Adjusted hazard ratio** | **Unadjusted hazard ratio** |
| **None** | **None** |
| **Adjusted odds ratio** | **Unadjusted odds ratio** |

1. **CRP**

| **** | **None** |
| --- | --- |
| **Adjusted hazard ratio** | **Unadjusted hazard ratio** |
| **None** | **** |
| **Adjusted odds ratio** | **Unadjusted odds ratio** |

1. **High CRP**

| **None** | **None** |
| --- | --- |
| **Adjusted hazard ratio** | **Unadjusted hazard ratio** |
| **None** | **None** |
| **Adjusted odds ratio** | **Unadjusted odds ratio** |

1. **ESR**

| **None** | **None** |
| --- | --- |
| **Adjusted hazard ratio** | **Unadjusted hazard ratio** |
| **None** | **None** |
| **Adjusted odds ratio** | **Unadjusted odds ratio** |

1. **High ESR**

| **None** | **None** |
| --- | --- |
| **Adjusted hazard ratio** | **Unadjusted hazard ratio** |
| **None** | **None** |
| **Adjusted odds ratio** | **Unadjusted odds ratio** |

1. **Albumin**

| **None** | **None** |
| --- | --- |
| **Adjusted hazard ratio** | **Unadjusted hazard ratio** |
| **None** | **None** |
| **Adjusted odds ratio** | **Unadjusted odds ratio** |

1. **Hypoalbuminemia**

| **None** | **None** |
| --- | --- |
| **Adjusted hazard ratio** | **Unadjusted hazard ratio** |
| **None** | **None** |
| **Adjusted odds ratio** | **Unadjusted odds ratio** |
