# Supplementary material for: The factors associated with mortality and progressive disease of nontuberculous mycobacterial lung disease: a systematic review and meta-analysis
Source: Sci Rep. 2023 May 5;13:7348. doi: 10.1038/s41598-023-34576-z (PMC10162985; doi:10.1038/s41598-023-34576-z)
Supplement: Supplementary file 8 — Supplementary Information 8. [file 41598_2023_34576_MOESM8_ESM.docx]

**Appendix S8. Pooled effect estimates having substantial heterogeneity**

| **Outcome: all-cause mortality** | | | |
| --- | --- | --- | --- |
| **Factors** | **Estimated effect size (95% confidence interval)** | **Number of included studies** | **I^2^ for heterogeneity** |
| Age | uHR 1.033 (0.976-1.094) | 2 | 77.8% |
| Body mass index | uOR 0.852 (0.706-1.029) | 2 | 68.6% |
| Chronic obstructive pulmonary disease | aHR 1.090 (0.373-3.183) | 2 | 55.3% |
| History of tuberculosis | uHR 1.519 (0.738-3.128) | 2 | 63.4% |
| History of tuberculosis | uOR 5.145 (0.352-75.268) | 2 | 80.1% |
| Chronic heart disease | aHR 1.959 (1.093-3.509) | 2 | 61.3% |
| Aspergillus co-infection | aHR 2.765 (0.760-10.063) | 2 | 54.7% |
| Cough | uHR 0.798 (0.232-2.749) | 2 | 88.1% |
| AFB smear positivity | uHR 1.719 (1.030-2.869) | 4 | 75.7% |
| Treatment duration | uHR 0.981 (0.907-1.062) | 2 | 84.1% |
| **Outcome: Clinical progressive disease with treatment** | | | |
| Elderly | uOR 0.745 (0.391-1.417) | 2 | 57.6% |
| Bronchiectatic pattern | uOR 0.520 (0.078-3.475) | 2 | 83.8% |
| Nodular-bronchiectatic pattern | uOR 0.502 (0.119-2.125) | 3 | 87.0% |
| AFB smear positivity | aHR 1.390 (0.934-2.069) | 3 | 71.6% |
| **Outcome: Radiographic progressive disease** | | | |
| Diabetes | aOR 0.971 (0.020-46.385) | 2 | 86.3% |
| Nodular-bronchiectatic pattern | uOR 0.136 (0.007-2.534) | 2 | 71.7% |
| High CRP | uOR 5.026 (0.870-29.054) | 2 | 68.2% |

Abbreviations: AFB, acid-fast bacillus; aHR, adjusted hazard ratio; aOR, adjusted odds ratio; CRP, C-reactive protein; uHR, unadjusted hazard ratio; uOR, unadjusted odds ratio
