# Supplementary material for: The factors associated with mortality and progressive disease of nontuberculous mycobacterial lung disease: a systematic review and meta-analysis
Source: Sci Rep. 2023 May 5;13:7348. doi: 10.1038/s41598-023-34576-z (PMC10162985; doi:10.1038/s41598-023-34576-z)
Supplement: Supplementary file 9 — Supplementary Information 9. [file 41598_2023_34576_MOESM9_ESM.docx]

**Appendix S9. Assessment of publication bias**

| **Outcome: all-cause mortality** | | | | | | | | | | | | | |  |
| --- | --- | --- | --- | --- | --- | --- | --- | --- | --- | --- | --- | --- | --- | --- |
| **Factors** | **Unadjusted HR** | **Begg’s test (*P*-value)** | **Egger's test (*P*-value)** | | **Adjusted HR** | **Begg’s test (*P*-value)** | **Egger's test (*P*-value)** | **Unadjusted OR** | **Begg’s test (*P*-value)** | **Egger's test (*P*-value)** | **Adjusted OR** | **Begg’s test (*P*-value)** | **Egger's test (*P*-value)** | |
| Age | 1.033 | 1.000 | - | | 1.052 | 0.230 | 0.049 | 1.050 | - | - | 1.030 | - | - | |
| Elderly | 1.408 | 1.000 | - | | 3.005 | 0.806 | 0.675 | - | - | - | - | - | - | |
| Male | 1.847 | 1.000 | 0.732 | | 2.406 | 0.474 | 0.595 | 2.287 | 1.000 | 0.604 | - | - | - | |
| Body mass index | 0.876 | 1.000 | - | | 0.832 | 0.452 | 0.375 | 0.852 | 1.000 | - | - | - | - | |
| Low body mass index | - | - | - | | 1.934 | 1.000 | 0.894 | - | - | - | - | - | - | |
| Ever-smoking | 2.666 | 0.734 | 0.492 | | - | - | - | 3.206 | - | - | - | - | - | |
| Any comorbidity | 1.347 | - | - | | 2.160 | - | - | - | - | - | - | - | - | |
| Diabetes | 2.471 | 1.000 | - | | 2.062 | 1.000 | 0.166 | 2.080 | 0.296 | 0.363 | - | - | - | |
| Chronic lung disease | 3.703 | 1.000 | - | | - | - | - | - | - | - | - | - | - | |
| Chronic obstructive pulmonary disease | 2.020 | - | - | | 1.090 | 1.000 | - | 2.137 | 0.296 | 0.418 | - | - | - | |
| History of tuberculosis | 1.519 | 1.000 | - | | 2.749 | 1.000 | - | 5.145 | 1.000 | - | - | - | - | |
| Bronchiectasis | 0.300 | - | - | | - | - | - | 0.379 | - | - | - | - | - | |
| Asthma | - | - | - | | - | - | - | 20.200 | - | - | - | - | - | |
| Interstitial lung disease | 1.830 | - | - | | - | - | - | 3.238 | - | - | - | - | - | |
| Pulmonary hypertension | - | - | - | | 2.100 | - | - | - | - | - | - | - | - | |
| Chronic heart disease | - | - | - | | 1.959 | 1.000 | - | 2.750 | - | - | - | - | - | |
| Chronic liver disease | 0.410 | - | - | | 1.86 | - | - | 2.815 | - | - | - | - | - | |
| Chronic kidney disease | 2.920 | - | - | | 1.701 | - | - | 5.963 | - | - | - | - | - | |
| Malignancy | - | - | - | | 2.213 | 0.806 | 0.290 | 1.988 | 1.000 | 0.752 | - | - | - | |
| Systemic immunosuppression | - | - | - | | 2.126 | 1.000 | - | 0.931 | 1.000 | - | - | - | - | |
| Aspergillus co-infection | - | - | - | | 2.765 | 1.000 | - | 1.275 | - | - | - | - | - | |
| Hemoptysis | 0.714 | 1.000 | - | | 0.542 | - | - | 0.849 | 1.000 | - | - | - | - | |
| Cough | 0.798 | 1.000 | - | | - | - | - | 0.692 | - | - | - | - | - | |
| Sputum | 1.490 | - | - | | - | - | - | 0.736 | - | - | - | - | - | |
| Fatigue | - | - | - | | - | - | - | 1.572 | - | - | - | - | - | |
| Dyspnea | 2.200 | - | - | | - | - | - | 1.615 | - | - | - | - | - | |
| Fever | - | - | - | | - | - | - | 0.810 | - | - | - | - | - | |
| Chest pain | - | - | - | | - | - | - | 0.496 | - | - | - | - | - | |
| Weight loss | - | - | - | | - | - | - | 3.433 | - | - | - | - | - | |
| Nodular pattern | - | - | - | | 1.830 | - | - | 1.110 | - | - | - | - | - | |
| Bronchiectatic pattern | 1.040 | - | - | | 0.573 | 1.000 | - | 0.560 | - | - | - | - | - | |
| Nodular-bronchiectatic pattern | 0.610 | - | - | | - | - | - | 0.432 | 1.000 | - | - | - | - | |
| Presence of cavity | 1.299 | - | - | | 2.380 | 0.049 | 0.031 | 1.101 | 1.000 | - | 3.176 | - | - | |
| Consolidative pattern | - | - | - | | 4.895 | 1.000 | - | - | - | - | - | - | - | |
| M.avium complex | 0.610 | - | - | | - | - | - | 0.678 | - | - | - | - | - | |
| M.kansasii | - | - | - | | - | - | - | 8.250 | - | - | - | - | - | |
| M.xenopi | - | - | - | | - | - | - | 0.800 | - | - | - | - | - | |
| AFB smear positivity | 1.719 | 0.734 | 0.462 | | 2.456 | 1.000 | - | 1.918 | - | - | - | - | - | |
| Rifamycin regimen | - | - | - | | 0.330 | - | - | - | - | - | - | - | - | |
| Treatment duration | 0.981 | 1.000 | - | | - | - | - | 0.917 | - | - | - | - | - | |
| Treatment with 3 or more antibiotics | - | - | - | | - | - | - | 0.865 | 1.000 | - | - | - | - | |
| Hb | 0.778 | - | - | | - | - | - | - | - | - | - | - | - | |
| Anemia | 2.320 | 1.000 | - | | 5.547 | - | - | - | - | - | - | - | - | |
| Platelet | - | - | - | | - | - | - | - | - | - | 1.090 | - | - | |
| CRP | 1.560 | - | - | | 1.220 | - | - | - | - | - | - | - | - | |
| High CRP | 3.614 | 1.000 | - | | 8.960 | - | - | - | - | - | - | - | - | |
| ESR | - | - | - | | 1.020 | 1.000 | - | - | - | - | - | - | - | |
| High ESR | - | - | - | | 1.849 | - | - | - | - | - | - | - | - | |
| Albumin | 0.286 | - | - | | - | - | - | - | - | - | - | - | - | |
| Hypoalbuminemia | - | - | - | | 3.770 | 1.000 | 0.986 | - | - | - | - | - | - | |
| **Outcome: Clinical progressive disease with treatment** | | | |  |  |  |  |  |  |  |  |  |  |  |
| **Factors** | **Unadjusted HR** | **Begg’s test (*P*-value)** | **Egger's test (*P*-value)** | | **Adjusted HR** | **Begg’s test (*P*-value)** | **Egger's test (*P*-value)** | **Unadjusted OR** | **Begg’s test (*P*-value)** | **Egger's test (*P*-value)** | **Adjusted OR** | **Begg’s test (*P*-value)** | **Egger's test (*P*-value)** | |
| Age | - | - | - | | 0.976 | 1.000 | 0.966 | - | - | - | 0.950 | - | - | |
| Elderly | - | - | - | | - | - | - | 0.745 | 1.000 | - | - | - | - | |
| Male | - | - | - | | 0.960 | - | - | 0.790 | 1.000 | 0.808 | - | - | - | |
| Body mass index | - | - | - | | 0.940 | - | - | 0.890 | - | - | - | - | - | |
| Low body mass index | - | - | - | | - | - | - | 4.250 | - | - | 0.515 | - | - | |
| Ever-smoking | - | - | - | | - | - | - | 1.104 | 1.000 | 0.264 | - | - | - | |
| Any comorbidity | - | - | - | | - | - | - | 0.653 | - | - | - | - | - | |
| Diabetes | - | - | - | | 1.340 | - | - | 0.716 | 1.000 | - | - | - | - | |
| Chronic lung disease | 1.000 | - | - | | - | - | - | 0.626 | 1.000 | - | - | - | - | |
| Chronic obstructive pulmonary disease | - | - | - | | 0.930 | - | - | 3.448 | 1.000 | - | 0.827 | - | - | |
| History of tuberculosis | - | - | - | | 1.230 | - | - | 1.177 | 0.734 | 0.299 | - | - | - | |
| Bronchiectasis | - | - | - | | - | - | - | 1.014 | 1.000 | - | - | - | - | |
| Interstitial lung disease | - | - | - | | - | - | - | 0.520 | 1.000 | - | - | - | - | |
| Chronic heart disease | - | - | - | | - | - | - | 0.701 | - | - | - | - | - | |
| Chronic liver disease | - | - | - | | - | - | - | 0.651 | - | - | - | - | - | |
| Chronic kidney disease | - | - | - | | - | - | - | 0.525 | - | - | - | - | - | |
| Malignancy | - | - | - | | 1.050 | - | - | 0.834 | 1.000 | 0.764 | - | - | - | |
| Systemic immunosuppression | 1.140 | - | - | | - | - | - | 0.916 | 1.000 | - | - | - | - | |
| Aspergillus co-infection | - | - | - | | - | - | - | - | - | - | 5.330 | - | - | |
| Hemoptysis | - | - | - | | 1.120 | - | - | 0.691 | 1.000 | - | 3.200 | - | - | |
| Cough | - | - | - | | 1.360 | - | - | 1.765 | 1.000 | - | 1.458 | - | - | |
| Sputum | - | - | - | | 1.470 | - | - | 0.775 | - | - | - | - | - | |
| Fatigue | - | - | - | | - | - | - | 2.940 | - | - | - | - | - | |
| Dyspnea | - | - | - | | - | - | - | 0.740 | 1.000 | - | - | - | - | |
| Fever | - | - | - | | - | - | - | 0.940 | - | - | - | - | - | |
| Weight loss | - | - | - | | - | - | - | - | - | - | 2.822 | 1.000 | - | |
| Nodular pattern | - | - | - | | - | - | - | 1.247 | 1.000 | - | - | - | - | |
| Bronchiectatic pattern | - | - | - | | - | - | - | 0.520 | 1.000 | - | - | - | - | |
| Nodular-bronchiectatic pattern | - | - | - | | - | - | - | 0.502 | 1.000 | 0.871 | - | - | - | |
| Presence of cavity | - | - | - | | 3.460 | - | - | 1.045 | 1.000 | - | 5.324 | 1.000 | - | |
| M.avium complex | - | - | - | | - | - | - | 0.670 | - | - | - | - | - | |
| M.abscessus | - | - | - | | - | - | - | 1.290 | - | - | - | - | - | |
| M.kansasii | - | - | - | | - | - | - | 3.110 | - | - | - | - | - | |
| M.xenopi | - | - | - | | - | - | - | 1.600 | - | - | - | - | - | |
| AFB smear positivity | - | - | - | | 1.390 | 0.296 | 0.535 | 2.078 | 1.000 | - | 2.132 | 1.000 | - | |
| WBC | - | - | - | | - | - | - | 1.950 | - | - | - | - | - | |
| Hb | - | - | - | | 0.890 | - | - | - | - | - | - | - | - | |
| CRP | - | - | - | | 1.000 | - | - | 2.700 | - | - | - | - | - | |
| **Outcome: Radiographic progressive disease** | | | | | | | | | | | | | | |
| **Factors** | **Unadjusted HR** | **Begg’s test (*P*-value)** | **Egger's test (*P*-value)** | | **Adjusted HR** | **Begg’s test (*P*-value)** | **Egger's test (*P*-value)** | **Unadjusted OR** | **Begg’s test (*P*-value)** | **Egger's test (*P*-value)** | **Adjusted OR** | **Begg’s test (*P*-value)** | **Egger's test (*P*-value)** | |
| Age | - | - | - | | 1.010 | - | - | 0.985 | - | - | 1.120 | - | - | |
| Elderly | - | - | - | | - | - | - | 1.778 | 1.000 | - | 2.980 | - | - | |
| Male | - | - | - | | 4.190 | - | - | 1.172 | 0.707 | 0.730 | - | - | - | |
| Body mass index | - | - | - | | 0.820 | - | - | 0.896 | 1.000 | - | 0.640 | - | - | |
| Low body mass index | - | - | - | | - | - | - | - | - | - | 0.930 | - | - | |
| Ever-smoking | 1.370 | - | - | | - | - | - | 1.307 | 1.000 | 0.871 | - | - | - | |
| Any comorbidity | - | - | - | | - | - | - | 0.339 | - | - | - | - | - | |
| Diabetes | 0.040 | - | - | | - | - | - | 0.433 | 1.000 | - | 0.971 | 1.000 | - | |
| Chronic obstructive pulmonary disease | 0.850 | - | - | | - | - | - | 1.822 | 1.000 | 0.182 | - | - | - | |
| History of tuberculosis | 1.590 | - | - | | - | - | - | 1.596 | 0.734 | 0.759 | 1.720 | - | - | |
| Bronchiectasis | - | - | - | | - | - | - | 1.172 | 1.000 | 0.986 | - | - | - | |
| Asthma | - | - | - | | 1.600 | - | - | 0.481 | 1.000 | - | - | - | - | |
| Interstitial lung disease | - | - | - | | 2.191 | - | - | 2.169 | 1.000 | - | - | - | - | |
| Chronic heart disease | - | - | - | | 6.190 | - | - | 0.625 | 1.000 | - | - | - | - | |
| Chronic liver disease | - | - | - | | - | - | - | 0.857 | - | - | - | - | - | |
| Chronic kidney disease | - | - | - | | - | - | - | 0.877 | - | - | - | - | - | |
| Malignancy | 0.040 | - | - | | - | - | - | 0.579 | 1.000 | - | - | - | - | |
| HIV | - | - | - | | 3.210 | - | - | 1.701 | - | - | - | - | - | |
| Systemic immunosuppression | 1.380 | - | - | | - | - | - | 1.854 | 1.000 | - | - | - | - | |
| Aspergillus co-infection | - | - | - | | - | - | - | 1.466 | - | - | - | - | - | |
| Hemoptysis | - | - | - | | - | - | - | 1.633 | 1.000 | - | - | - | - | |
| Cough | - | - | - | | - | - | - | 0.717 | 1.000 | - | - | - | - | |
| Sputum | - | - | - | | - | - | - | 1.107 | 1.000 | - | - | - | - | |
| Dyspnea | - | - | - | | - | - | - | 0.903 | - | - | - | - | - | |
| Fever | - | - | - | | - | - | - | 1.254 | - | - | - | - | - | |
| Chest pain | - | - | - | | - | - | - | 0.363 | - | - | - | - | - | |
| Weight loss | - | - | - | | - | - | - | 0.923 | - | - | - | - | - | |
| Nodular pattern | - | - | - | | - | - | - | 0.495 | - | - | - | - | - | |
| Bronchiectatic pattern | - | - | - | | - | - | - | 0.944 | - | - | - | - | - | |
| Nodular-bronchiectatic pattern | - | - | - | | - | - | - | 0.136 | 1.000 | - | - | - | - | |
| Presence of cavity | - | - | - | | 1.651 | - | - | 2.655 | 1.000 | - | 3.283 | 1.000 | - | |
| Consolidative pattern | - | - | - | | - | - | - | 1.778 | - | - | 16.150 | - | - | |
| M.avium complex | - | - | - | | - | - | - | 0.680 | - | - | - | - | - | |
| M.abscessus | - | - | - | | - | - | - | - | - | - | 1.360 | - | - | |
| AFB smear positivity | - | - | - | | 3.380 | - | - | 1.315 | 0.296 | 0.218 | 1.852 | - | - | |
| Treatment with 3 or more antibiotics | - | - | - | | - | - | - | 0.786 | - | - | - | - | - | |
| Leukocytosis | - | - | - | | - | - | - | - | - | - | 3.440 | - | - | |
| Anemia | - | - | - | | 1.852 | - | - | 1.518 | - | - | - | - | - | |
| Thrombocytopenia | - | - | - | | - | - | - | 1.770 | - | - | - | - | - | |
| CRP | - | - | - | | - | - | - | 0.925 | - | - | - | - | - | |
| High CRP | - | - | - | | 1.520 | - | - | 5.026 | 1.000 | - | - | - | - | |
| ESR | - | - | - | | - | - | - | - | - | - | 1.010 | - | - | |
| Albumin | - | - | - | | - | - | - | 1.456 | - | - | - | - | - | |
| Hypoalbuminemia | - | - | - | | - | - | - | 2.898 | 1.000 | - | - | - | - | |

Abbreviations: AFB, acid-fast bacillus; CRP, C-reactive protein; ESR, erythrocyte sedimentation rate; HIV, human immunodeficiency virus; HR, hazard ratio; OR, odds ratio; WBC, white blood cell
