# Supplementary material for: The factors associated with mortality and progressive disease of nontuberculous mycobacterial lung disease: a systematic review and meta-analysis
Source: Sci Rep. 2023 May 5;13:7348. doi: 10.1038/s41598-023-34576-z (PMC10162985; doi:10.1038/s41598-023-34576-z)
Supplement: Supplementary file 10 — Supplementary Information 10. [file 41598_2023_34576_MOESM10_ESM.docx]

**Appendix S10. Assessment of certainty of evidence of prognostic factors by the Grades of Recommendation, Assessment,**

**Development and Evaluation (GRADE) system**

| **Outcome: all-cause mortality** | | | | | | | | | | |
| --- | --- | --- | --- | --- | --- | --- | --- | --- | --- | --- |
| **Factors** | **Estimated effect size**  **(95% confidence interval)** | **Phase** | **Study limitations** | **Inconsistency** | **Indirectness** | **Imprecision** | **Publication bias** | **Moderate/large effect size** | **Dose effect** | **Overall quality** |
| Age | aHR 1.052 (1.031-1.074) | 1 | $\times$ | ✓ | $\times$ | ✓ | $\times$ | $\times$ | $\times$ | + |
| Age | uHR 1.033 (0.976-1.094) | 1 | $\times$ | $\times$ | $\times$ | $\times$ | ✓ | $\times$ | $\times$ | + |
| Age | aOR 1.030 (0.997-1.064) | 1 | $\times$ | N/A | $\times$ | $\times$ | ✓ | $\times$ | $\times$ | + |
| Age | uOR 1.050 (0.998-1.105) | 1 | $\times$ | N/A | ✓ | $\times$ | ✓ | $\times$ | $\times$ | + |
| Elderly | aHR 3.005 (2.329-3.876) | 1 | $\times$ | ✓ | $\times$ | ✓ | ✓ | ✓ | $\times$ | ++ |
| Elderly | uHR 1.408 (0.898-2.209) | 1 | $\times$ | ✓ | $\times$ | $\times$ | ✓ | $\times$ | $\times$ | + |
| Male | aHR 2.406 (1.900-3.047) | 1 | $\times$ | ✓ | $\times$ | ✓ | ✓ | $\times$ | $\times$ | + |
| Male | uHR 1.847 (1.078-3.164) | 1 | $\times$ | ✓ | $\times$ | $\times$ | ✓ | $\times$ | $\times$ | + |
| Male | uOR 2.287 (1.379-3.794) | 1 | $\times$ | ✓ | $\times$ | $\times$ | ✓ | $\times$ | $\times$ | + |
| Body mass index | aHR 0.832 (0.788-0.878) | 1 | $\times$ | ✓ | $\times$ | ✓ | ✓ | $\times$ | $\times$ | + |
| Body mass index | uHR 0.876 (0.799-0.959) | 1 | $\times$ | ✓ | $\times$ | $\times$ | ✓ | $\times$ | $\times$ | + |
| Body mass index | uOR 0.852 (0.706-1.029) | 1 | $\times$ | $\times$ | ✓ | $\times$ | ✓ | $\times$ | $\times$ | + |
| Low body mass index | aHR 1.934 (1.581-2.366) | 1 | ✓ | ✓ | $\times$ | ✓ | ✓ | $\times$ | $\times$ | ++ |
| Ever-smoking | uHR 2.666 (2.111-3.369) | 1 | $\times$ | ✓ | $\times$ | ✓ | ✓ | ✓ | $\times$ | ++ |
| Ever-smoking | uOR 3.206 (1.140-9.014) | 1 | $\times$ | N/A | ✓ | $\times$ | ✓ | ✓ | $\times$ | + |
| Any comorbidity | aHR 2.160 (0.957-4.875) | 1 | $\times$ | N/A | ✓ | $\times$ | ✓ | $\times$ | $\times$ | + |
| Any comorbidity | uHR 1.347 (0.451-4.023) | 1 | $\times$ | N/A | $\times$ | $\times$ | ✓ | $\times$ | $\times$ | + |
| Diabetes | aHR 2.062 (1.194-3.562) | 1 | $\times$ | ✓ | $\times$ | ✓ | ✓ | $\times$ | $\times$ | + |
| Diabetes | uHR 2.471 (1.863-3.278) | 1 | ✓ | ✓ | ✓ | $\times$ | ✓ | $\times$ | $\times$ | ++ |
| Diabetes | uOR 2.080 (0.981-4.412) | 1 | $\times$ | ✓ | $\times$ | $\times$ | ✓ | $\times$ | $\times$ | + |
| Chronic lung disease | uHR 3.703 (2.490-5.508) | 1 | $\times$ | ✓ | $\times$ | $\times$ | ✓ | ✓ | $\times$ | + |
| Chronic obstructive pulmonary disease | aHR 1.090 (0.373-3.183) | 1 | $\times$ | $\times$ | ✓ | $\times$ | ✓ | $\times$ | $\times$ | + |
| Chronic obstructive pulmonary disease | uHR 2.020 (0.670-6.088) | 1 | $\times$ | N/A | ✓ | $\times$ | ✓ | $\times$ | $\times$ | + |
| Chronic obstructive pulmonary disease | uOR 2.137 (1.194-3.823) | 1 | $\times$ | ✓ | $\times$ | $\times$ | ✓ | $\times$ | $\times$ | + |
| History of tuberculosis | aHR 2.749 (1.341-5.637) | 1 | $\times$ | ✓ | ✓ | $\times$ | ✓ | ✓ | $\times$ | ++ |
| History of tuberculosis | uHR 1.519 (0.738-3.128) | 1 | ✓ | $\times$ | ✓ | $\times$ | ✓ | $\times$ | $\times$ | + |
| History of tuberculosis | uOR 5.145 (0.352-75.268) | 1 | $\times$ | $\times$ | $\times$ | $\times$ | ✓ | ✓ | $\times$ | + |
| Bronchiectasis | uHR 0.300 (0.090-0.995) | 1 | $\times$ | N/A | $\times$ | $\times$ | ✓ | $\times$ | $\times$ | + |
| Bronchiectasis | uOR 0.379 (0.147-0.977) | 1 | $\times$ | N/A | $\times$ | $\times$ | ✓ | $\times$ | $\times$ | + |
| Asthma | uOR 20.200 (3.366-121.216) | 1 | $\times$ | N/A | ✓ | $\times$ | ✓ | ✓ | $\times$ | + |
| Interstitial lung disease | uHR 1.830 (0.844-3.967) | 1 | $\times$ | N/A | $\times$ | $\times$ | ✓ | $\times$ | $\times$ | + |
| Interstitial lung disease | uOR 3.238 (0.887-11.820) | 1 | $\times$ | N/A | $\times$ | $\times$ | ✓ | ✓ | $\times$ | + |
| Pulmonary hypertension | aHR 2.100 (0.882-4.999) | 1 | $\times$ | N/A | ✓ | $\times$ | ✓ | $\times$ | $\times$ | + |
| Chronic heart disease | aHR 1.959 (1.093-3.509) | 1 | ✓ | $\times$ | ✓ | $\times$ | ✓ | $\times$ | $\times$ | + |
| Chronic heart disease | uOR 2.750 (0.649-11.648) | 1 | $\times$ | N/A | $\times$ | $\times$ | ✓ | ✓ | $\times$ | + |
| Chronic liver disease | aHR 1.860 (1.242-2.785) | 1 | ✓ | N/A | ✓ | $\times$ | ✓ | $\times$ | $\times$ | + |
| Chronic liver disease | uHR 0.410 (0.100-1.679) | 1 | $\times$ | N/A | $\times$ | $\times$ | ✓ | $\times$ | $\times$ | + |
| Chronic liver disease | uOR 2.815 (0.792-10.004) | 1 | $\times$ | N/A | $\times$ | $\times$ | ✓ | ✓ | $\times$ | + |
| Chronic kidney disease | aHR 1.701 (0.685-4.225) | 1 | $\times$ | N/A | $\times$ | $\times$ | ✓ | $\times$ | $\times$ | + |
| Chronic kidney disease | uHR 2.920 (0.919-9.279) | 1 | $\times$ | N/A | $\times$ | $\times$ | ✓ | ✓ | $\times$ | + |
| Chronic kidney disease | uOR 5.963 (1.613-22.042) | 1 | $\times$ | N/A | $\times$ | $\times$ | ✓ | ✓ | $\times$ | + |
| Malignancy | aHR 2.213 (1.680-2.914) | 1 | $\times$ | ✓ | $\times$ | ✓ | ✓ | $\times$ | $\times$ | + |
| Malignancy | uOR 1.988 (0.992-3.985) | 1 | $\times$ | ✓ | $\times$ | $\times$ | ✓ | $\times$ | $\times$ | + |
| Systemic immunosuppression | aHR 2.126 (1.311-3.450) | 1 | $\times$ | ✓ | $\times$ | $\times$ | ✓ | $\times$ | $\times$ | + |
| Systemic immunosuppression | uOR 0.931 (0.313-2.769) | 1 | $\times$ | ✓ | $\times$ | $\times$ | ✓ | $\times$ | $\times$ | + |
| Aspergillus co-infection | aHR 2.765 (0.760-10.063) | 1 | ✓ | $\times$ | ✓ | $\times$ | ✓ | ✓ | $\times$ | ++ |
| Aspergillus co-infection | uOR 1.275 (0.730-2.225) | 1 | $\times$ | ✓ | ✓ | $\times$ | ✓ | $\times$ | $\times$ | + |
| Hemoptysis | aHR 0.542 (0.414-0.709) | 1 | $\times$ | N/A | $\times$ | $\times$ | ✓ | $\times$ | $\times$ | + |
| Hemoptysis | uHR 0.714 (0.542-0.940) | 1 | ✓ | ✓ | ✓ | $\times$ | ✓ | $\times$ | $\times$ | ++ |
| Hemoptysis | uOR 0.849 (0.281-2.567) | 1 | $\times$ | ✓ | $\times$ | $\times$ | ✓ | $\times$ | $\times$ | + |
| Cough | uHR 0.798 (0.232-2.749) | 1 | ✓ | $\times$ | ✓ | $\times$ | ✓ | $\times$ | $\times$ | + |
| Cough | uOR 0.692 (0.257-1.865) | 1 | $\times$ | N/A | $\times$ | $\times$ | ✓ | $\times$ | $\times$ | + |
| Sputum | uHR 1.490 (1.097-2.024) | 1 | ✓ | N/A | ✓ | $\times$ | ✓ | $\times$ | $\times$ | + |
| Sputum | uOR 0.736 (0.344-1.574) | 1 | $\times$ | N/A | $\times$ | $\times$ | ✓ | $\times$ | $\times$ | + |
| Fatigue | uOR 1.572 (0.714-3.460) | 1 | $\times$ | N/A | $\times$ | $\times$ | ✓ | $\times$ | $\times$ | + |
| Dyspnea | uHR 2.200 (0.924-5.237) | 1 | $\times$ | N/A | $\times$ | $\times$ | ✓ | $\times$ | $\times$ | + |
| Dyspnea | uOR 1.615 (0.754-3.461) | 1 | $\times$ | N/A | $\times$ | $\times$ | ✓ | $\times$ | $\times$ | + |
| Fever | uOR 0.810 (0.261-2.513) | 1 | $\times$ | N/A | $\times$ | $\times$ | ✓ | $\times$ | $\times$ | + |
| Chest pain | uOR 0.496 (0.109-2.253) | 1 | $\times$ | N/A | $\times$ | $\times$ | ✓ | $\times$ | $\times$ | + |
| Weight loss | uOR 3.433 (1.587-7.425) | 1 | $\times$ | N/A | $\times$ | $\times$ | ✓ | ✓ | $\times$ | + |
| Nodular pattern | aHR 1.830 (0.491-6.815) | 1 | $\times$ | N/A | ✓ | $\times$ | ✓ | $\times$ | $\times$ | + |
| Nodular pattern | uOR 1.110 (0.518-2.380) | 1 | $\times$ | N/A | $\times$ | $\times$ | ✓ | $\times$ | $\times$ | + |
| Bronchiectatic pattern | aHR 0.573 (0.245-1.339) | 1 | $\times$ | ✓ | ✓ | $\times$ | ✓ | $\times$ | $\times$ | + |
| Bronchiectatic pattern | uHR 1.040 (0.661-1.637) | 1 | $\times$ | N/A | $\times$ | $\times$ | ✓ | $\times$ | $\times$ | + |
| Bronchiectatic pattern | uOR 0.560 (0.251-1.250) | 1 | $\times$ | N/A | $\times$ | $\times$ | ✓ | $\times$ | $\times$ | + |
| Nodular-bronchiectatic pattern | uHR 0.610 (0.299-1.244) | 1 | $\times$ | N/A | $\times$ | $\times$ | ✓ | $\times$ | $\times$ | + |
| Nodular-bronchiectatic pattern | uOR 0.432 (0.216-0.862) | 1 | $\times$ | ✓ | ✓ | $\times$ | ✓ | $\times$ | $\times$ | + |
| Presence of cavity | aHR 2.380 (1.866-3.037) | 1 | $\times$ | ✓ | $\times$ | ✓ | ✓ | $\times$ | $\times$ | + |
| Presence of cavity | uHR 1.299 (0.751-2.247) | 1 | $\times$ | N/A | $\times$ | $\times$ | $\times$ | $\times$ | $\times$ | + |
| Presence of cavity | aOR 3.176 (1.369-7.369) | 1 | $\times$ | N/A | $\times$ | $\times$ | ✓ | ✓ | $\times$ | + |
| Presence of cavity | uOR 1.101 (0.395-3.069) | 1 | $\times$ | ✓ | ✓ | $\times$ | ✓ | $\times$ | $\times$ | + |
| Consolidative pattern | aHR 4.895 (2.997-7.996) | 1 | $\times$ | ✓ | ✓ | $\times$ | ✓ | ✓ | $\times$ | ++ |
| M.avium complex | uHR 0.610 (0.202-1.839) | 1 | $\times$ | N/A | ✓ | $\times$ | ✓ | $\times$ | $\times$ | + |
| M.avium complex | uOR 0.678 (0.457-1.005) | 1 | $\times$ | ✓ | ✓ | $\times$ | ✓ | $\times$ | $\times$ | + |
| M.kansasii | uOR 8.250 (0.492-138.363) | 1 | $\times$ | N/A | ✓ | $\times$ | ✓ | ✓ | $\times$ | + |
| M.xenopi | uOR 0.800 (0.388-1.650) | 1 | $\times$ | N/A | $\times$ | $\times$ | ✓ | $\times$ | $\times$ | + |
| AFB smear positivity | aHR 2.456 (1.460-4.130) | 1 | $\times$ | ✓ | ✓ | $\times$ | ✓ | $\times$ | $\times$ | + |
| AFB smear positivity | uHR 1.719 (1.030-2.869) | 1 | $\times$ | $\times$ | $\times$ | ✓ | ✓ | $\times$ | $\times$ | + |
| AFB smear positivity | uOR 1.918 (0.670-5.489) | 1 | $\times$ | N/A | ✓ | $\times$ | ✓ | $\times$ | $\times$ | + |
| Rifamycin regimen | aHR 0.330 (0.151-0.723) | 1 | $\times$ | N/A | $\times$ | $\times$ | ✓ | $\times$ | $\times$ | + |
| Treatment duration | uHR 0.981 (0.907-1.062) | 1 | $\times$ | $\times$ | ✓ | $\times$ | ✓ | $\times$ | $\times$ | + |
| Treatment duration | uOR 0.917 (0.864-0.973) | 1 | $\times$ | N/A | $\times$ | $\times$ | ✓ | $\times$ | $\times$ | + |
| Treatment with 3 or more antibiotics | uOR 0.865 (0.454-1.648) | 1 | $\times$ | ✓ | $\times$ | $\times$ | ✓ | $\times$ | $\times$ | + |
| Hb | uHR 0.778 (0.629-0.962) | 1 | $\times$ | N/A | $\times$ | $\times$ | ✓ | $\times$ | $\times$ | + |
| Anemia | aHR 5.547 (1.235-24.916) | 1 | $\times$ | N/A | $\times$ | $\times$ | ✓ | ✓ | $\times$ | + |
| Anemia | uHR 2.320 (1.377-3.909) | 1 | $\times$ | ✓ | $\times$ | $\times$ | ✓ | $\times$ | $\times$ | + |
| Platelet | aOR 1.090 (1.008-1.178) | 1 | $\times$ | N/A | ✓ | $\times$ | ✓ | $\times$ | $\times$ | + |
| CRP | aHR 1.220 (1.058-1.407) | 1 | $\times$ | N/A | $\times$ | $\times$ | ✓ | $\times$ | $\times$ | + |
| CRP | uHR 1.560 (1.321-1.843) | 1 | $\times$ | N/A | $\times$ | $\times$ | ✓ | $\times$ | $\times$ | + |
| High CRP | aHR 8.960 (1.657-48.462) | 1 | $\times$ | N/A | $\times$ | $\times$ | ✓ | ✓ | $\times$ | + |
| High CRP | uHR 3.614 (2.296-5.688) | 1 | $\times$ | ✓ | $\times$ | $\times$ | ✓ | ✓ | $\times$ | + |
| ESR | aHR 1.020 (1.016-1.024) | 1 | ✓ | ✓ | $\times$ | $\times$ | ✓ | $\times$ | $\times$ | + |
| High ESR | aHR 1.849 (1.140-2.999) | 1 | $\times$ | N/A | $\times$ | $\times$ | ✓ | $\times$ | $\times$ | + |
| Albumin | uHR 0.286 (0.172-0.474) | 1 | $\times$ | N/A | $\times$ | $\times$ | ✓ | $\times$ | $\times$ | + |
| Hypoalbuminemia | aHR 3.770 (2.697-5.270) | 1 | $\times$ | ✓ | $\times$ | ✓ | ✓ | ✓ | $\times$ | ++ |
| **Outcome: Clinical progressive disease with treatment** | | | | | | | | | | |
| **Factors** | **Estimated effect size**  **(95% confidence interval)** | **Phase** | **Study limitations** | **Inconsistency** | **Indirectness** | **Imprecision** | **Publication bias** | **Moderate/large effect size** | **Dose effect** | **Overall quality** |
| Age | aHR 0.976 (0.967-0.985) | 1 | $\times$ | ✓ | $\times$ | ✓ | ✓ | $\times$ | $\times$ | + |
| Age | aOR 0.950 (0.920-0.980) | 1 | $\times$ | N/A | $\times$ | $\times$ | ✓ | $\times$ | $\times$ | + |
| Elderly | uOR 0.745 (0.391-1.417) | 1 | $\times$ | $\times$ | ✓ | $\times$ | ✓ | $\times$ | $\times$ | + |
| Male | aHR 0.960 (0.451-2.044) | 1 | $\times$ | N/A | $\times$ | $\times$ | ✓ | $\times$ | $\times$ | + |
| Male | uOR 0.790 (0.582-1.072) | 1 | $\times$ | ✓ | ✓ | ✓ | ✓ | $\times$ | $\times$ | ++ |
| Body mass index | aHR 0.940 (0.872-1.013) | 1 | ✓ | N/A | ✓ | $\times$ | ✓ | $\times$ | $\times$ | + |
| Body mass index | uOR 0.890 (0.795-0.996) | 1 | ✓ | N/A | $\times$ | $\times$ | ✓ | $\times$ | $\times$ | + |
| Low body mass index | aOR 0.515 (0.310-0.855) | 1 | $\times$ | N/A | $\times$ | $\times$ | ✓ | $\times$ | $\times$ | + |
| Low body mass index | uOR 4.250 (1.184-15.253) | 1 | $\times$ | N/A | ✓ | $\times$ | ✓ | ✓ | $\times$ | ++ |
| Ever-smoking | uOR 1.104 (0.716-1.701) | 1 | $\times$ | ✓ | $\times$ | ✓ | ✓ | $\times$ | $\times$ | + |
| Any comorbidity | uOR 0.653 (0.195-2.188) | 1 | $\times$ | N/A | $\times$ | $\times$ | ✓ | $\times$ | $\times$ | + |
| Diabetes | aHR 1.340 (0.938-1.915) | 1 | $\times$ | N/A | $\times$ | $\times$ | ✓ | $\times$ | $\times$ | + |
| Diabetes | uOR 0.716 (0.429-1.195) | 1 | $\times$ | ✓ | ✓ | $\times$ | ✓ | $\times$ | $\times$ | + |
| Chronic lung disease | uHR 1.000 (0.656-1.524) | 1 | $\times$ | N/A | $\times$ | $\times$ | ✓ | $\times$ | $\times$ | + |
| Chronic lung disease | uOR 0.626 (0.241-1.629) | 1 | $\times$ | ✓ | $\times$ | $\times$ | ✓ | ✓ | $\times$ | + |
| Chronic obstructive pulmonary disease | aHR 0.930 (0.717-1.206) | 1 | $\times$ | N/A | $\times$ | $\times$ | ✓ | $\times$ | $\times$ | + |
| Chronic obstructive pulmonary disease | aOR 0.827 (0.410-1.669) | 1 | $\times$ | N/A | $\times$ | $\times$ | ✓ | $\times$ | $\times$ | + |
| Chronic obstructive pulmonary disease | uOR 3.448 (1.906-6.238) | 1 | $\times$ | ✓ | ✓ | $\times$ | ✓ | ✓ | $\times$ | ++ |
| History of tuberculosis | aHR 1.230 (1.009-1.499) | 1 | $\times$ | N/A | $\times$ | $\times$ | ✓ | $\times$ | $\times$ | + |
| History of tuberculosis | uOR 1.177 (0.843-1.644) | 1 | $\times$ | ✓ | ✓ | ✓ | ✓ | $\times$ | $\times$ | ++ |
| Bronchiectasis | uOR 1.014 (0.514-2.001) | 1 | $\times$ | ✓ | ✓ | $\times$ | ✓ | $\times$ | $\times$ | + |
| Interstitial lung disease | uOR 0.520 (0.160-1.695) | 1 | $\times$ | ✓ | ✓ | $\times$ | ✓ | $\times$ | $\times$ | + |
| Chronic heart disease | uOR 0.701 (0.201-2.448) | 1 | $\times$ | N/A | $\times$ | $\times$ | ✓ | $\times$ | $\times$ | + |
| Chronic liver disease | uOR 0.651 (0.349-1.215) | 1 | $\times$ | N/A | $\times$ | $\times$ | ✓ | $\times$ | $\times$ | + |
| Chronic kidney disease | uOR 0.525 (0.116-2.372) | 1 | $\times$ | N/A | $\times$ | $\times$ | ✓ | $\times$ | $\times$ | + |
| Malignancy | aHR 1.050 (0.742-1.485) | 1 | $\times$ | N/A | $\times$ | $\times$ | ✓ | $\times$ | $\times$ | + |
| Malignancy | uOR 0.834 (0.449-1.546) | 1 | $\times$ | ✓ | ✓ | ✓ | ✓ | $\times$ | $\times$ | ++ |
| Systemic immunosuppression | uHR 1.140 (0.609-2.133) | 1 | $\times$ | N/A | $\times$ | $\times$ | ✓ | $\times$ | $\times$ | + |
| Systemic immunosuppression | uOR 0.916 (0.462-1.815) | 1 | $\times$ | ✓ | ✓ | $\times$ | ✓ | $\times$ | $\times$ | + |
| Aspergillus co-infection | aOR 5.330 (1.107-25.662) | 1 | $\times$ | N/A | ✓ | $\times$ | ✓ | ✓ | $\times$ | + |
| Hemoptysis | aHR 1.120 (0.913-1.374) | 1 | $\times$ | N/A | $\times$ | $\times$ | ✓ | $\times$ | $\times$ | + |
| Hemoptysis | aOR 3.200 (0.281-36.434) | 1 | $\times$ | N/A | ✓ | $\times$ | ✓ | ✓ | $\times$ | + |
| Hemoptysis | uOR 0.691 (0.486-0.983) | 1 | $\times$ | ✓ | ✓ | $\times$ | ✓ | $\times$ | $\times$ | + |
| Cough | aHR 1.360 (1.053-1.756) | 1 | $\times$ | N/A | $\times$ | $\times$ | ✓ | $\times$ | $\times$ | + |
| Cough | aOR 1.458 (0.923-2.303) | 1 | $\times$ | N/A | $\times$ | $\times$ | ✓ | $\times$ | $\times$ | + |
| Cough | uOR 1.765 (0.799-3.899) | 1 | $\times$ | ✓ | ✓ | $\times$ | ✓ | $\times$ | $\times$ | + |
| Sputum | aHR 1.470 (1.131-1.911) | 1 | $\times$ | N/A | $\times$ | $\times$ | ✓ | $\times$ | $\times$ | + |
| Sputum | uOR 0.775 (0.552-1.089) | 1 | $\times$ | N/A | $\times$ | $\times$ | ✓ | $\times$ | $\times$ | + |
| Fatigue | uOR 2.940 (0.907-9.532) | 1 | $\times$ | N/A | ✓ | $\times$ | ✓ | ✓ | $\times$ | + |
| Dyspnea | uOR 0.740 (0.472-1.161) | 1 | $\times$ | ✓ | ✓ | $\times$ | ✓ | $\times$ | $\times$ | + |
| Fever | uOR 0.940 (0.283-3.118) | 1 | $\times$ | N/A | ✓ | $\times$ | ✓ | $\times$ | $\times$ | + |
| Weight loss | aOR 2.822 (1.271-6.268) | 1 | $\times$ | ✓ | ✓ | $\times$ | ✓ | ✓ | $\times$ | ++ |
| Nodular pattern | uOR 1.247 (0.554-2.809) | 1 | $\times$ | ✓ | ✓ | $\times$ | ✓ | $\times$ | $\times$ | + |
| Bronchiectatic pattern | uOR 0.520 (0.078-3.475) | 1 | $\times$ | $\times$ | ✓ | $\times$ | ✓ | $\times$ | $\times$ | + |
| Nodular-bronchiectatic pattern | uOR 0.502 (0.119-2.125) | 1 | $\times$ | $\times$ | $\times$ | ✓ | ✓ | $\times$ | $\times$ | + |
| Presence of cavity | aHR 3.460 (2.273-5.267) | 1 | $\times$ | N/A | $\times$ | $\times$ | ✓ | ✓ | $\times$ | + |
| Presence of cavity | aOR 5.324 (2.323-12.199) | 1 | $\times$ | ✓ | ✓ | $\times$ | ✓ | ✓ | $\times$ | ++ |
| Presence of cavity | uOR 1.045 (0.358-3.051) | 1 | $\times$ | ✓ | $\times$ | $\times$ | ✓ | ✓ | $\times$ | + |
| M.avium complex | uOR 0.670 (0.300-1.498) | 1 | $\times$ | N/A | ✓ | $\times$ | ✓ | $\times$ | $\times$ | + |
| M.abscessus | uOR 1.290 (0.226-7.369) | 1 | $\times$ | N/A | ✓ | $\times$ | ✓ | $\times$ | $\times$ | + |
| M.kansasii | uOR 3.110 (1.030-9.390) | 1 | $\times$ | N/A | ✓ | $\times$ | ✓ | ✓ | $\times$ | + |
| M.xenopi | uOR 1.600 (0.309-8.289) | 1 | $\times$ | N/A | ✓ | $\times$ | ✓ | $\times$ | $\times$ | + |
| AFB smear positivity | aHR 1.390 (0.934-2.069) | 1 | $\times$ | $\times$ | $\times$ | ✓ | ✓ | $\times$ | $\times$ | + |
| AFB smear positivity | aOR 2.132 (1.393-3.263) | 1 | $\times$ | ✓ | $\times$ | $\times$ | ✓ | $\times$ | $\times$ | + |
| AFB smear positivity | uOR 2.078 (1.148-3.762) | 1 | $\times$ | ✓ | ✓ | $\times$ | ✓ | $\times$ | $\times$ | + |
| WBC | uOR 1.950 (0.867-4.385) | 1 | $\times$ | N/A | ✓ | $\times$ | ✓ | $\times$ | $\times$ | + |
| Hb | aHR 0.890 (0.749-1.058) | 1 | ✓ | N/A | ✓ | $\times$ | ✓ | $\times$ | $\times$ | ++ |
| CRP | aHR 1.000 (0.970-1.030) | 1 | $\times$ | N/A | $\times$ | $\times$ | ✓ | $\times$ | $\times$ | + |
| CRP | uOR 2.700 (1.179-6.184) | 1 | $\times$ | N/A | ✓ | $\times$ | ✓ | ✓ | $\times$ | + |
| **Outcome: Radiographic progressive disease** | | | | | | | | | | |
| **Factors** | **Estimated effect size**  **(95% confidence interval)** | **Phase** | **Study limitations** | **Inconsistency** | **Indirectness** | **Imprecision** | **Publication bias** | **Moderate/large effect size** | **Dose effect** | **Overall quality** |
| Age | aHR 1.010 (0.971-1.051) | 1 | $\times$ | N/A | $\times$ | $\times$ | ✓ | $\times$ | $\times$ | + |
| Age | aOR 1.120 (1.038-1.209) | 1 | $\times$ | N/A | $\times$ | $\times$ | ✓ | $\times$ | $\times$ | + |
| Age | uOR 0.985 (0.957-1.013) | 1 | $\times$ | N/A | $\times$ | $\times$ | ✓ | $\times$ | $\times$ | + |
| Elderly | aOR 2.980 (1.041-8.534) | 1 | $\times$ | N/A | $\times$ | $\times$ | ✓ | ✓ | $\times$ | + |
| Elderly | uOR 1.778 (0.835-3.787) | 1 | $\times$ | ✓ | ✓ | $\times$ | ✓ | $\times$ | $\times$ | + |
| Male | aHR 4.190 (0.929-18.898) | 1 | $\times$ | N/A | $\times$ | $\times$ | ✓ | $✓$ | $\times$ | + |
| Male | uOR 1.172 (0.752-1.826) | 1 | $\times$ | ✓ | $\times$ | ✓ | ✓ | $\times$ | $\times$ | + |
| Body mass index | aHR 0.820 (0.668-1.007) | 1 | ✓ | N/A | $\times$ | $\times$ | ✓ | $\times$ | $\times$ | + |
| Body mass index | aOR 0.640 (0.370-1.109) | 1 | $\times$ | N/A | $\times$ | $\times$ | ✓ | $\times$ | $\times$ | + |
| Body mass index | uOR 0.896 (0.815-0.986) | 1 | $\times$ | ✓ | $\times$ | $\times$ | ✓ | $\times$ | $\times$ | + |
| Low body mass index | aOR 0.930 (0.281-3.074) | 1 | $\times$ | N/A | ✓ | $\times$ | ✓ | $\times$ | $\times$ | + |
| Ever-smoking | uHR 1.370 (0.362-5.187) | 1 | $\times$ | N/A | $\times$ | $\times$ | ✓ | $\times$ | $\times$ | + |
| Ever-smoking | uOR 1.307 (0.712-2.400) | 1 | $\times$ | ✓ | $\times$ | ✓ | ✓ | $\times$ | $\times$ | + |
| Any comorbidity | uOR 0.339 (0.141-0.815) | 1 | $\times$ | N/A | ✓ | $\times$ | ✓ | $\times$ | $\times$ | + |
| Diabetes | uHR 0.040 (0.000-43.644) | 1 | $\times$ | N/A | $\times$ | $\times$ | ✓ | $\times$ | $\times$ | + |
| Diabetes | aOR 0.971 (0.020-46.385) | 1 | $\times$ | $\times$ | $\times$ | $\times$ | ✓ | $\times$ | $\times$ | + |
| Diabetes | uOR 0.433 (0.146-1.288) | 1 | $\times$ | ✓ | ✓ | $\times$ | ✓ | $\times$ | $\times$ | + |
| Chronic obstructive pulmonary disease | uHR 0.850 (0.240-3.005) | 1 | $\times$ | N/A | $\times$ | $\times$ | ✓ | $\times$ | $\times$ | + |
| Chronic obstructive pulmonary disease | uOR 1.822 (0.922-3.604) | 1 | $\times$ | ✓ | $\times$ | ✓ | ✓ | ✓ | $\times$ | + |
| History of tuberculosis | uHR 1.590 (0.448-5.639) | 1 | $\times$ | N/A | $\times$ | $\times$ | ✓ | $\times$ | $\times$ | + |
| History of tuberculosis | aOR 1.720 (0.490-6.034) | 1 | $\times$ | N/A | ✓ | $\times$ | ✓ | $\times$ | $\times$ | + |
| History of tuberculosis | uOR 1.596 (0.969-2.628) | 1 | $\times$ | ✓ | $\times$ | $\times$ | ✓ | $\times$ | $\times$ | + |
| Bronchiectasis | uOR 1.172 (0.590-2.328) | 1 | $\times$ | ✓ | $\times$ | ✓ | ✓ | $\times$ | $\times$ | + |
| Asthma | aHR 1.600 (0.190-13.466) | 1 | $\times$ | N/A | $\times$ | $\times$ | ✓ | $\times$ | $\times$ | + |
| Asthma | uOR 0.481 (0.112-2.071) | 1 | $\times$ | ✓ | $\times$ | $\times$ | ✓ | $\times$ | $\times$ | + |
| Interstitial lung disease | aHR 2.191 (1.325-3.623) | 1 | $\times$ | N/A | $\times$ | $\times$ | ✓ | $\times$ | $\times$ | + |
| Interstitial lung disease | uOR 2.169 (0.554-8.488) | 1 | $\times$ | ✓ | $\times$ | $\times$ | ✓ | $\times$ | $\times$ | + |
| Chronic heart disease | aHR 6.190 (0.170-225.374) | 1 | $\times$ | N/A | $\times$ | $\times$ | ✓ | ✓ | $\times$ | + |
| Chronic heart disease | uOR 0.625 (0.245-1.594) | 1 | $\times$ | ✓ | $\times$ | $\times$ | ✓ | $\times$ | $\times$ | + |
| Chronic liver disease | uOR 0.857 (0.164-4.476) | 1 | $\times$ | N/A | $\times$ | $\times$ | ✓ | $\times$ | $\times$ | + |
| Chronic kidney disease | uOR 0.877 (0.266-2.892) | 1 | $\times$ | N/A | $\times$ | $\times$ | ✓ | $\times$ | $\times$ | + |
| Malignancy | uHR 0.040 (0.000-122.171) | 1 | $\times$ | N/A | $\times$ | $\times$ | ✓ | $\times$ | $\times$ | + |
| Malignancy | uOR 0.579 (0.103-3.269) | 1 | $\times$ | ✓ | $\times$ | $\times$ | ✓ | $\times$ | $\times$ | + |
| HIV | aHR 3.210 (0.562-18.334) | 1 | $\times$ | N/A | $\times$ | $\times$ | ✓ | ✓ | $\times$ | + |
| HIV | uOR 1.701 (0.171-16.927) | 1 | $\times$ | N/A | $\times$ | $\times$ | ✓ | $\times$ | $\times$ | + |
| Systemic immunosuppression | uHR 1.380 (0.390-4.886) | 1 | $\times$ | N/A | $\times$ | $\times$ | ✓ | $\times$ | $\times$ | + |
| Systemic immunosuppression | uOR 1.854 (0.455-7.562) | 1 | $\times$ | ✓ | $\times$ | $\times$ | ✓ | $\times$ | $\times$ | + |
| Aspergillus co-infection | uOR 1.466 (0.772-2.784) | 1 | $\times$ | N/A | ✓ | $\times$ | ✓ | $\times$ | $\times$ | + |
| Hemoptysis | uOR 1.633 (0.643-4.148) | 1 | $\times$ | ✓ | $\times$ | $\times$ | ✓ | $\times$ | $\times$ | + |
| Cough | uOR 0.717 (0.356-1.443) | 1 | $\times$ | ✓ | $\times$ | $\times$ | ✓ | $\times$ | $\times$ | + |
| Sputum | uOR 1.107 (0.498-2.460) | 1 | $\times$ | ✓ | $\times$ | $\times$ | ✓ | $\times$ | $\times$ | + |
| Dyspnea | uOR 0.903 (0.406-2.008) | 1 | $\times$ | N/A | $\times$ | $\times$ | ✓ | $\times$ | $\times$ | + |
| Fever | uOR 1.254 (0.435-3.614) | 1 | $\times$ | N/A | $\times$ | $\times$ | ✓ | $\times$ | $\times$ | + |
| Chest pain | uOR 0.363 (0.116-1.137) | 1 | $\times$ | N/A | $\times$ | $\times$ | ✓ | $\times$ | $\times$ | + |
| Weight loss | uOR 0.923 (0.208-4.092) | 1 | $\times$ | N/A | $\times$ | $\times$ | ✓ | $\times$ | $\times$ | + |
| Nodular pattern | uOR 0.495 (0.220-1.113) | 1 | $\times$ | N/A | $\times$ | $\times$ | ✓ | $\times$ | $\times$ | + |
| Bronchiectatic pattern | uOR 0.944 (0.407-2.192) | 1 | $\times$ | N/A | $\times$ | $\times$ | ✓ | $\times$ | $\times$ | + |
| Nodular-bronchiectatic pattern | uOR 0.136 (0.007-2.534) | 1 | $\times$ | $\times$ | $\times$ | $\times$ | ✓ | $\times$ | $\times$ | + |
| Presence of cavity | aHR 1.651 (1.181-2.308) | 1 | ✓ | N/A | $\times$ | ✓ | ✓ | $\times$ | $\times$ | + |
| Presence of cavity | aOR 3.283 (1.405-7.673) | 1 | $\times$ | ✓ | ✓ | $\times$ | ✓ | ✓ | $\times$ | ++ |
| Presence of cavity | uOR 2.655 (1.209-5.831) | 1 | $\times$ | ✓ | $\times$ | $\times$ | ✓ | ✓ | $\times$ | + |
| Consolidative pattern | aOR 16.150 (4.048-64.429) | 1 | $\times$ | N/A | $\times$ | $\times$ | ✓ | ✓ | $\times$ | + |
| Consolidative pattern | uOR 1.778 (0.331-9.552) | 1 | $\times$ | N/A | $\times$ | $\times$ | ✓ | $\times$ | $\times$ | + |
| M.avium complex | uOR 0.680 (0.273-1.693) | 1 | $\times$ | N/A | ✓ | $\times$ | ✓ | $\times$ | $\times$ | + |
| M.abscessus | aOR 1.360 (0.291-6.354) | 1 | $\times$ | N/A | ✓ | $\times$ | ✓ | $\times$ | $\times$ | + |
| AFB smear positivity | aHR 3.380 (0.830-13.767) | 1 | $\times$ | N/A | $\times$ | $\times$ | ✓ | $✓$ | $\times$ | + |
| AFB smear positivity | aOR 1.852 (0.745-4.605) | 1 | $\times$ | N/A | $\times$ | $\times$ | ✓ | $\times$ | $\times$ | + |
| AFB smear positivity | uOR 1.315 (0.764-2.265) | 1 | $\times$ | ✓ | $\times$ | ✓ | ✓ | $\times$ | $\times$ | + |
| Treatment with 3 or more antibiotics | uOR 0.786 (0.188-3.288) | 1 | $\times$ | N/A | $\times$ | $\times$ | ✓ | $\times$ | $\times$ | + |
| Leukocytosis | aOR 3.440 (1.139-10.390) | 1 | $\times$ | N/A | $\times$ | $\times$ | ✓ | ✓ | $\times$ | + |
| Anemia | aHR 1.852 (1.265-2.712) | 1 | ✓ | N/A | $\times$ | $\times$ | ✓ | $\times$ | $\times$ | + |
| Anemia | uOR 1.518 (0.692-3.329) | 1 | $\times$ | N/A | $\times$ | $\times$ | ✓ | $\times$ | $\times$ | + |
| Thrombocytopenia | uOR 1.770 (0.450-6.965) | 1 | $\times$ | N/A | $\times$ | $\times$ | ✓ | $\times$ | $\times$ | + |
| CRP | uOR 0.925 (0.782-1.095) | 1 | $\times$ | N/A | $\times$ | $\times$ | ✓ | $\times$ | $\times$ | + |
| High CRP | aHR 1.520 (1.081-2.137) | 1 | ✓ | N/A | $\times$ | $\times$ | ✓ | $\times$ | $\times$ | + |
| High CRP | uOR 5.026 (0.870-29.054) | 1 | $\times$ | $\times$ | $\times$ | $\times$ | ✓ | ✓ | $\times$ | + |
| ESR | aOR 1.010 (0.929-1.098) | 1 | $\times$ | N/A | $\times$ | $\times$ | ✓ | $\times$ | $\times$ | + |
| Albumin | uOR 1.456 (0.839-2.528) | 1 | $\times$ | N/A | $\times$ | $\times$ | ✓ | $\times$ | $\times$ | + |
| Hypoalbuminemia | uOR 2.898 (1.143-7.347) | 1 | $\times$ | ✓ | $\times$ | $\times$ | ✓ | ✓ | $\times$ | + |

Abbreviations: AFB, acid-fast bacillus; aHR, adjusted hazard ratio; aOR, adjusted odds ratio; CRP, C-reactive protein; ESR, erythrocyte sedimentation rate; HIV, human immunodeficiency virus; uHR, unadjusted hazard ratio; uOR, unadjusted odds ratio

✓, no serious limitations; $\times$, serious limitations (or not present for moderate/large effect size, dose effect); N/A, not applicable. For overall quality of evidence: +, very low; ++, low; +++, moderate; ++++, high
