# Supplementary material for: The factors associated with mortality and progressive disease of nontuberculous mycobacterial lung disease: a systematic review and meta-analysis
Source: Sci Rep. 2023 May 5;13:7348. doi: 10.1038/s41598-023-34576-z (PMC10162985; doi:10.1038/s41598-023-34576-z)
Supplement: Supplementary file 11 — Supplementary Information 11. [file 41598_2023_34576_MOESM11_ESM.docx]

**Appendix S11. Search strategy for the systematic review and meta-analysis**

**PubMed**

#1. Non-tuberculous mycobacteria

“Non-tuberculous*”[Title] OR “Nontuberculous*”[Title] OR NTM[Title] OR “Mycobacterium abcessus”[Title] OR “Mycobacterium avium”[Title] OR “Mycobacterium avium Complex”[Title] OR “Mycobacterium chelonae”[Title] OR “Mycobacterium chimaera”[Title] OR “Mycobacterium fortuitum”[Title] OR “Mycobacterium intracellulare”[Title] OR “Mycobacterium kansasii”[Title] OR “Mycobacterium malmoense”[Title] OR “Mycobacterium massiliense”[Title] OR “Mycobacterium mucogenicum”[Title] OR “Mycobacterium simiae”[Title] OR “Mycobacterium xenopi”[Title]

OR

“Mycobacterium Infections, Nontuberculous”[mesh] OR “Nontuberculous Mycobacteria”[mesh] OR “Mycobacterium abscessus”[mesh] OR “Mycobacterium avium”[mesh] OR “Mycobacterium avium Complex”[mesh] OR “Mycobacterium avium-intracellulare Infection”[mesh] OR “Mycobacterium chelonae”[mesh] OR “Mycobacterium chimaera”[Supplementary Concept] OR “Mycobacterium fortuitum”[mesh] OR “Mycobacterium kansasii”[mesh] OR “Mycobacterium malmoense”[Supplementary Concept] OR “Mycolicibacterium mucogenicum”[Supplementary Concept] OR “Mycobacterium simiae”[Supplementary Concept] OR “Mycobacterium xenopi”[mesh]

#2. Lung disease

Lung[Title] OR Pulmonary[Title]

#3. Outcomes

Progress*[tiab] OR Deterioration[tiab]

OR “Disease progression”[Mesh]

OR

Mortality[tiab] OR Death[tiab] OR Surviv*[tiab]

OR “Mortality”[Mesh] OR “Death”[Mesh] OR “Survival”[Mesh]

OR

Prognos*[tiab] OR Predict*[tiab]

OR “Prognosis”[Mesh]

#1 AND #2 AND #3

**N= 493 (2021.4.12)**

**EMBASE**

#1. Non-tuberculous mycobacteria

‘Non-tuberculous mycobacteria*’:ti OR ‘Nontuberculous mycobacteria*’:ti OR NTM:ti OR ‘Mycobacterium abcessus’:ti OR ‘Mycobacterium avium’:ti OR ‘Mycobacterium avium Complex’:ti OR ‘Mycobacterium chelonae’:ti OR ‘Mycobacterium chimaera’:ti OR ‘Mycobacterium fortuitum’:ti OR ‘Mycobacterium intercellulare’:ti OR ‘Mycobacterium kansasii’:ti OR ‘Mycobacterium malmoense’:ti OR ‘Mycobacterium massiliense’:ti OR ‘Mycobacterium mucogenicum’:ti OR ‘Mycobacterium simiae’:ti OR ‘Mycobacterium xenopi’:ti

OR

'atypical mycobacteriosis'/exp OR ‘Mycobacterium abscessus’/exp OR ‘Mycobacterium avium’/exp OR ‘Mycobacterium avium complex’/exp OR ‘Mycobacterium chelonae’/exp OR ‘Mycobacterium chimaera’/exp OR ‘Mycobacterium fortuitum’/exp OR ‘Mycobacterium kansasii’/exp OR ‘Mycobacterium malmoense’/exp OR ‘Mycobacterium massiliense’/exp OR ‘Mycobacterium mucogenicum’/exp OR ‘Mycobacterium simiae’/exp OR ‘Mycobacterium xenopi’/exp

#2. Lung disease

Lung:ti OR Pulmonary:ti

#3. Outcomes

Progress*:ab,ti OR Deterioration:ab,ti

OR ‘disease exacerbation’/exp

OR

Mortality:ab,ti OR Death:ab,ti OR Surviv*:ab,ti

OR ‘mortality’/exp OR ‘death’/exp OR ‘survival’/exp

OR

Prognos*:ab,ti OR Predict*:ab,ti

OR ‘prognosis’/exp

#1 AND #2 AND #3

**N= 848 (2021.4.12)**

**Cochrane library**

#1. Non-tuberculous mycobacteria

“Nontuberculous mycobacteria*”:ti OR NTM:ti OR “Mycobacterium abcessus”:ti OR “Mycobacterium avium”:ti OR “Mycobacterium avium Complex”:ti OR “Mycobacterium chelonae”:ti OR “Mycobacterium chimaera”:ti OR “Mycobacterium fortuitum”:ti OR “Mycobacterium intercellulare”:ti OR “Mycobacterium kansasii”:ti OR “Mycobacterium malmoense”:ti OR “Mycobacterium massiliense”:ti OR “Mycobacterium mucogenicum”:ti OR “Mycobacterium simiae”:ti OR “Mycobacterium xenopi”:ti

OR

[mh “Mycobacterium Infections, Nontuberculous”] OR [mh “Nontuberculous Mycobacteria”] OR [mh “Mycobacterium abscessus”] OR [mh “Mycobacterium avium”] OR [mh “Mycobacterium avium Complex”] OR [mh “Mycobacterium avium-intracellulare Infection”] OR [mh “Mycobacterium chelonae”] OR [mh “Mycobacterium fortuitum”] OR [mh “Mycobacterium kansasii”] OR [mh “Mycobacterium xenopi”]

#2. Lung disease

Lung:ti OR Pulmonary:ti

#3. Outcomes

Progress*:ti,ab,kw OR Deterioration:ti,ab,kw

OR [mh “Disease progression”]

OR

Mortality:ti,ab,kw OR Death:ti,ab,kw OR Surviv*:ti,ab,kw

OR [mh “Mortality”] OR [mh “Death”] OR [mh “Survival”]

OR

Prognos*:ti,ab,kw OR Predict*:ti,ab,kw

OR [mh “Prognosis”]

#1 AND #2 AND #3

**N= 19 (2021.4.12)**

**Web of science**

#1. Non-tuberculous mycobacteria

TI = “Non-tuberculous mycobacteria*” OR “Nontuberculous mycobacteria*” OR NTM OR “Mycobacterium abcessus” OR “Mycobacterium avium” OR “Mycobacterium avium Complex” OR “Mycobacterium chelonae” OR “Mycobacterium chimaera” OR “Mycobacterium fortuitum” OR “Mycobacterium intercellulare” OR “Mycobacterium kansasii” OR “Mycobacterium malmoense” OR “Mycobacterium massiliense” OR “Mycobacterium mucogenicum” OR “Mycobacterium simiae” OR “Mycobacterium xenopi”

#2. Lung disease

TI = Lung OR Pulmonary

#3. Outcomes

TS = Progress* OR Deterioration OR Mortality OR Death OR Surviv* OR Prognos* OR Predict*

#1 AND #2 AND #3

**N= 288 (2021.4.12)**
