# Supplementary figures and images for: The factors associated with mortality and progressive disease of nontuberculous mycobacterial lung disease: a systematic review and meta-analysis
Source: Sci Rep. 2023 May 5;13:7348. doi: 10.1038/s41598-023-34576-z (PMC10162985; doi:10.1038/s41598-023-34576-z)

a)

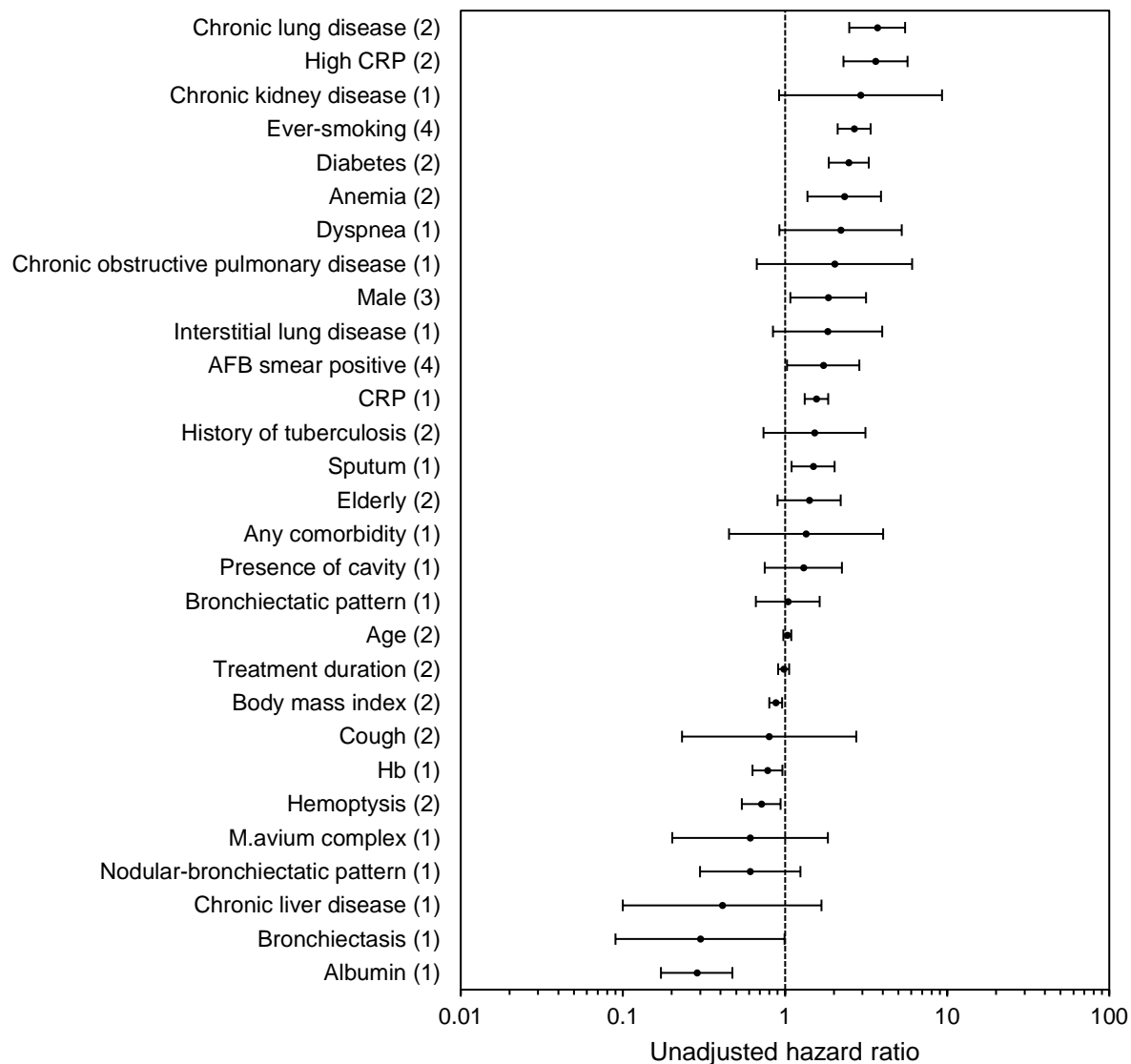

b)

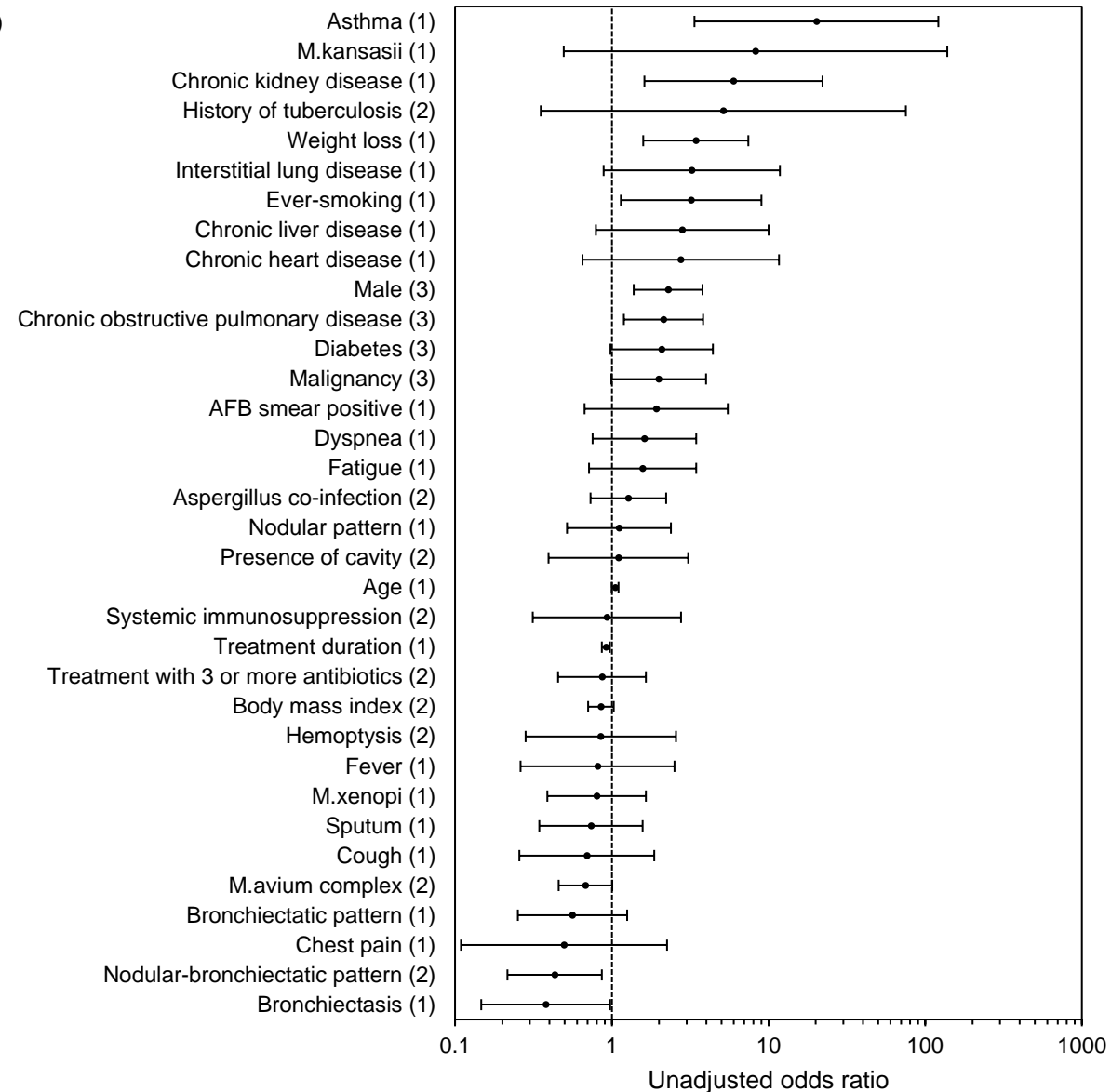

Supplement: Supplementary file 12 — Supplementary Information 12. [file 41598_2023_34576_MOESM12_ESM.pdf]

a)

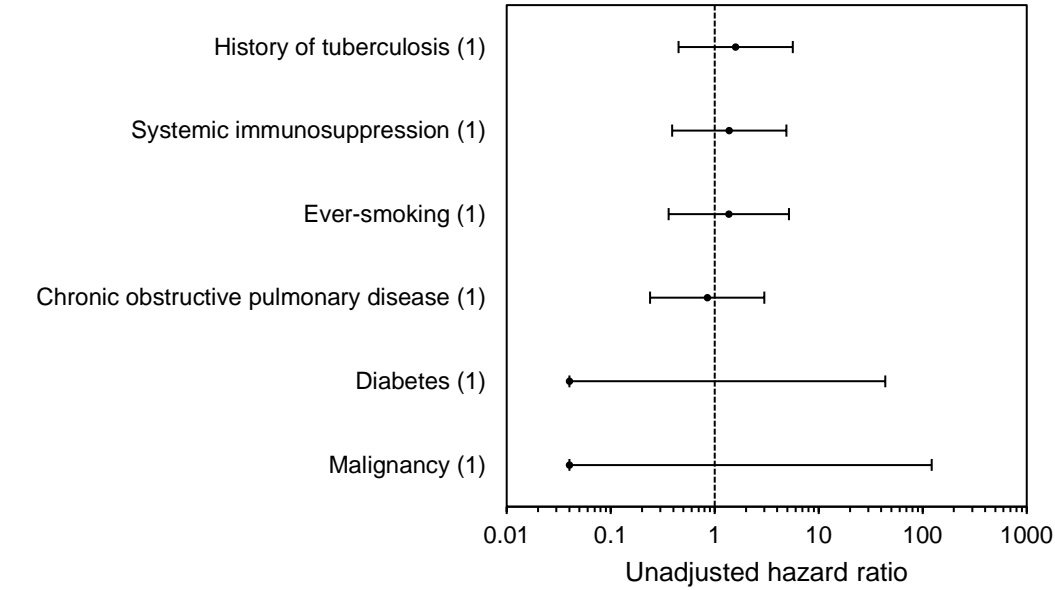

b)

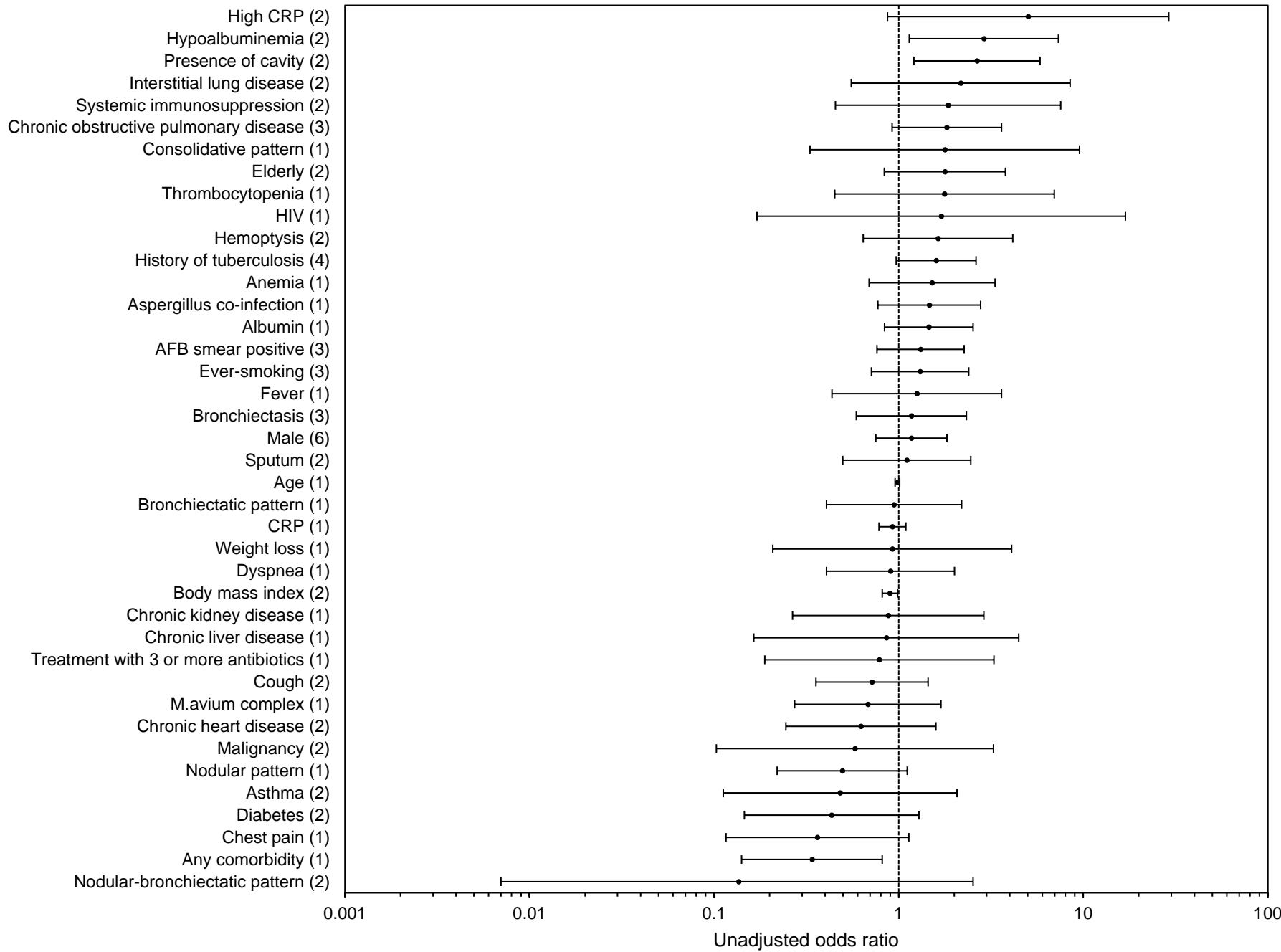

Supplement: Supplementary file 14 — Supplementary Information 14. [file 41598_2023_34576_MOESM14_ESM.pdf]
