# Supplementary material for: The factors associated with mortality and progressive disease of nontuberculous mycobacterial lung disease: a systematic review and meta-analysis
Source: Sci Rep. 2023 May 5;13:7348. doi: 10.1038/s41598-023-34576-z (PMC10162985; doi:10.1038/s41598-023-34576-z)
Supplement: Supplementary file 13 — Supplementary Information 13. [file 41598_2023_34576_MOESM13_ESM.pdf]

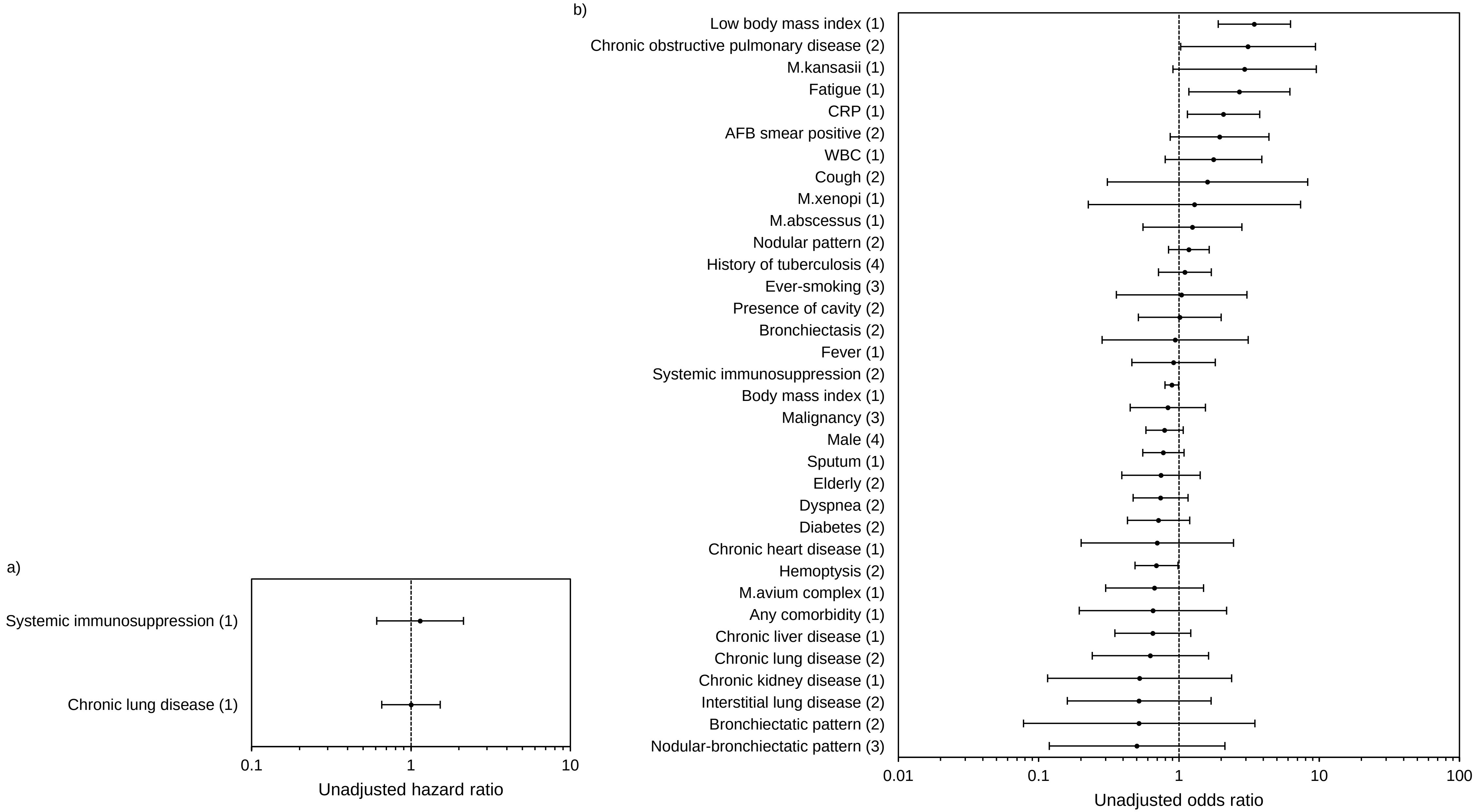
a)
Systemic immunosuppression (1)
Chronic lung disease (1)
0.1
1
10
Unadjusted hazard ratio
b)
Low body mass index (1)
Chronic obstructive pulmonary disease (2)
M.kansasii (1)
Fatigue (1)
CRP (1)
AFB smear positive (2)
WBC (1)
Cough (2)
M.xenopi (1)
M.abscessus (1)
Nodular pattern (2)
History of tuberculosis (4)
Ever-smoking (3)
Presence of cavity (2)
Bronchiectasis (2)
Fever (1)
Systemic immunosuppression (2)
Body mass index (1)
Malignancy (3)
Male (4)
Sputum (1)
Elderly (2)
Dyspnea (2)
Diabetes (2)
Chronic heart disease (1)
Hemoptysis (2)
M.avium complex (1)
Any comorbidity (1)
Chronic liver disease (1)
Chronic lung disease (2)
Chronic kidney disease (1)
Interstitial lung disease (2)
Bronchiectatic pattern (2)
Nodular-bronchiectatic pattern (3)
0.01
0.1
1
10
100
Unadjusted odds ratio
